# Axial Phenoxylation of Aluminum Phthalocyanines for Improved Cannabinoid Sensitivity in OTFT Sensors

**DOI:** 10.1002/advs.202305515

**Published:** 2024-04-19

**Authors:** Halynne R. Lamontagne, Rosemary R. Cranston, Zachary J. Comeau, Cory S. Harris, Adam J. Shuhendler, Benoît H. Lessard

**Affiliations:** ^1^ Department of Chemical and Biological Engineering University of Ottawa 161 Louis Pasteur Ottawa ON K1N 6N5 Canada; ^2^ Department of Chemistry and Biomolecular Sciences University of Ottawa 150 Louis Pasteur Ottawa ON K1N 6N5 Canada; ^3^ Advanced Electronics and Devices National Research Council Canada 1200 Montreal Rd Ottawa ON K1A 0R6 Canada; ^4^ Department of Biology University of Ottawa 30 Marie Curie Ottawa ON K1N 6N5 Canada; ^5^ University of Ottawa Heart Institute 40 Ruskin St Ottawa ON K1Y 4W7 Canada; ^6^ School of Electrical Engineering and Computer Science University of Ottawa 800 King Edward Ave Ottawa ON K1N 6N5 Canada

**Keywords:** aluminum phthalocyanine, cannabinoid sensor, grazing‐incidence wide‐angle X‐ray scattering, organic thin‐film transistor, thin‐film engineering

## Abstract

Cannabis producers, consumers, and regulators need fast, accurate, point‐of‐use sensors to detect Δ^9^‐tetrahydrocannabinol (THC) and cannabidiol (CBD) from both liquid and vapor source samples, and phthalocyanine‐based organic thin‐film transistors (OTFTs) provide a cost‐effective solution. Chloro aluminum phthalocyanine (Cl‐AlPc) has emerged as a promising material due to its unique coordinating interactions with cannabinoids, allowing for superior sensitivity. This work explores the molecular engineering of AlPc to tune and enhance these interactions, where a series of novel phenxoylated R‐AlPcs are synthesized and integrated into OTFTs, which are then exposed to THC and CBD solution and vapor samples. While the R‐AlPc substituted molecules have a comparable baseline device performance to Cl‐AlPc, their new crystal structures and weakened intermolecular interactions increase sensitivity to THC. Grazing‐incidence wide‐angle X‐ray scattering (GIWAXS) and atomic force microscopy (AFM) are used to investigate this film restructuring, where a significant shift in the crystal structure, grain size, and film roughness is detected for the R‐AlPc molecules that do not occur with Cl‐AlPc. This significant crystal reorganization and film restructuring are the driving force behind the improved sensitivity to cannabinoids relative to Cl‐AlPc and demonstrate that analyte–semiconductor interactions can be enhanced through chemical modification to create more responsive OTFT sensors.

## Graphical abstract

Phenoxy‐aluminum phthalocyanine is a promising semiconductor material that can be used in organic thin‐film transistor (OTFT) based sensors for the detection of Δ^9^‐tetrahydrocannabinol (THC) and cannabidiol (CBD). Structure–property relationships are established between the choice of axial groups and the thin film restructuring that takes place as the driving force behind the improved sensitivity to both vapor and solution cannabinoids.

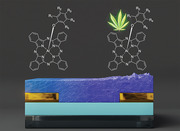

## 1 Introduction

As countries around the world move to legalize cannabis, there is a growing burden on producers and regulators to accurately measure and monitor the quality of cannabis products for their cannabinoid content. The two major cannabinoids, Δ^9^‐tetrahydrocannabinol (THC) and cannabidiol (CBD), have different but specific psychogenic and therapeutic effects on the human body when they are consumed.^[^ ^1^, ^2^, ^3^, ^4^, ^5^ ^]^ They are also chemically similar, making them difficult to differentiate and measure in samples.^[^ ^2^, ^6^ ^]^ Currently in industry, this differentiation is often performed by high‐performance liquid chromatography (HPLC), an expensive and time‐consuming technique that requires trained personnel.^[^ ^7^, ^8^ ^]^ There is a present need for quick, accurate, point‐of‐source tests to identify and speciate THC and CBD in a variety of source samples. A demonstrated potential low‐cost solution to this problem is the use of phthalocyanine‐based organic thin‐film transistors (OTFTs).^[^ ^9^, ^10^, ^11^, ^12^, ^13^ ^]^

Phthalocyanines (Pcs) are conjugated macrocyclic organic compounds which can chelate a variety of metals and metalloids (MPcs). Their chemical stability, alongside unique spectral and electronic properties, has led to Pc applications as dyes and pigments,^[^ ^14^, ^15^ ^]^ and as charge transport layers in OTFTs^[^ ^16^, ^17^, ^18^, ^19^, ^20^, ^21^, ^22^ ^]^ and organic photovoltaics (OPVs).^[^ ^16^, ^23^, ^24^, ^25^ ^]^ They are inexpensive to synthesize and can easily be chemically modified through peripheral substituents,^[^ ^26^, ^27^, ^28^, ^29^ ^]^ axial substituents^[^ ^18^, ^30^, ^31^, ^32^, ^33^, ^34^, ^35^, ^36^, ^37^, ^38^ ^]^ or substituting the central metal.^[^ ^39^ ^]^ This structural versatility enables the tuning of their physical properties to achieve greater solubility, new crystal polymorphs, desirable film morphology, or improved electronic properties.^[^ ^14^, ^40^, ^41^, ^42^ ^]^ For example, the choice of axial groups in silicon phthalocyanines (R_2_‐SiPc)^[^ ^43^ ^]^ has enabled the fine tuning of solubility/miscibility,^[^ ^44^ ^]^ processability,^[^ ^17^ ^]^ morphology,^[^ ^18^, ^35^, ^36^ ^]^ and OTFT performance such as threshold voltage^[^ ^37^, ^38^, ^45^ ^]^ or air stability.^[^ ^29^, ^46^ ^]^ Pc‐based OTFTs have also been used for a variety of liquid and gas sensing applications,^[^ ^19^, ^47^, ^48^, ^49^, ^50^ ^]^ including as cannabinoid sensors to ratiometrically detect and differentiate THC and CBD in plant and vapor samples.^[^ ^9^, ^10^, ^11^, ^12^, ^13^ ^]^ Our group's previous work has identified that this sensing response arises from a combination of electrochemical and physical interactions between cannabinoids and thin‐films of Pcs.^[^ ^10^, ^11^, ^12^, ^13^ ^]^ These interactions lead to a structural change in the Pc film through induced surface crystallization, causing a change in the electrical performance of the OTFT.^[^ ^11^, ^12^, ^13^ ^]^ The magnitude and nature of these structural changes varies depending on the MPc, which highlights the importance of both the choice of Pc, and its thin‐film structure for the overall performance of an OTFT sensor.

Chloro aluminum phthalocyanine (Cl‐AlPc) has demonstrated superior performance as a cannabinoid sensor in previous work.^[^ ^10^, ^12^ ^]^ Cl‐AlPc interacts strongly with cannabinoids both in solution and as a thin‐film, leading to a greater change in electrical response.^[^ ^10^, ^12^ ^]^ The large aluminum atom and the single chloro axial substituent of Cl‐AlPc causes the molecule to form an inverted umbrella shape, which exposes its inner ring of nitrogen to interactions with analytes.^[^ ^10^ ^]^ Cl‐AlPc also results in a relatively loose crystal packing structure compared to other divalent MPcs.^[^ ^51^ ^]^ This open crystal packing can be exploited by switching the Cl‐ atom for a larger pendant group, changing the crystal structure and further exposing the inner nitrogen ring, potentially leading to more sensitive devices. Phenoxylation is a facile reaction which can be performed on axial halogens of MPcs and which has proven effective at engineering crystals and improving solubility in boron subphthalocyanines (BsubPcs),^[^ ^24^, ^26^, ^52^ ^]^ a class of MPcs which also have an inverted umbrella molecular shape with a single axial group. Several phenoxylated BsubPcs have emerged in literature where even subtle changes to the phenoxy group, such as a well‐placed methyl group or halogen group can lead to significant changes in solubility and crystal orientation, respectively.^[^ ^53^, ^54^ ^]^ While relatively less explored, the phenoxylation of Cl‐AlPc can be carried out under similar conditions^[^ ^55^ ^]^ and could therefore benefit from such crystal engineering.

**Figure 1:**
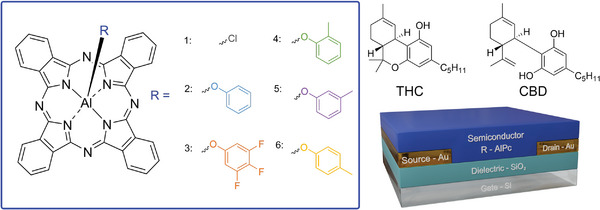
Schematic diagram of the axial phenoxy‐AlPcs used as semiconductors in a bottom‐gate bottom‐contact (BGBC) OTFT cannabinoid sensor, including the structure of THC and CBD. 1: Chloro aluminum phthalocyanine (Cl‐AlPc); 2: phenoxy aluminum phthalocyanine (PhO‐AlPc); 3: 3,4,5‐trifluorophenoxy aluminum phthalocyanine (345F‐AlPc); 4: 2‐methylphenoxy aluminum phthalocyanine (oCr‐AlPc); 5: 3‐methylphenoxy aluminum phthalocyanine (mCr‐AlPc); 6: 4‐methylphenoxy aluminum phthalocyanine (pCr‐AlPc).

Here, we synthesized a series of novel trifluoro‐ and monomethylphenoxylated aluminum phthalocyanine derivatives (R‐AlPc) and integrated them into OTFTs as the semiconducting layer for the first time (**Figure** **1**). We characterized these R‐AlPc compounds in terms of their response to THC and CBD in OTFT based sensors. Grazing‐incidence wide‐angle X‐ray scattering (GIWAXS) and atomic force microscopy (AFM) were used to probe the changes in thin film structure which led to improved sensor performance. Thus, we demonstrate phenoxylation as a straightforward technique to alter the thin film structure and sensitize axially substituted Pc‐based OTFTs to analytes without compromising performance.

## 2 Results and Discussion

### 2.1 Axial Phenoxylation of Aluminum Phthalocyanines

A series of axial phenoxylation coupling reactions were performed on Cl‐AlPc by reaction with different phenols at 10‐molar excess in chlorobenzene at 125 °C, as outlined by Raboui et al. (Figure S1, Supporting Information).^[^ ^55^ ^]^ The reaction conditions and resulting yields for all substitutions performed can be found in Table S1 (Supporting Information). The chosen synthesized compounds include phenoxy‐AlPc (PhO‐AlPc), 3,4,5‐trifluorophenoxy‐AlPc (345F‐AlPc), 2‐methylphenoxy‐AlPc (oCr‐AlPc), 3‐methylphenoxy‐AlPc (mCr‐AlPc), and 4‐methylphenoxy‐AlPc (pCr‐AlPc). These substitutions were chosen to study both the effect of fluorination and methyl position on AlPc film morphology and resulting sensor performance, as both have been found to greatly influence Pc solubility and electrical performance.^[^ ^29^, ^38^, ^55^, ^56^, ^57^ ^]^ Other substitutions were attempted to expand the arsenal of phenoxy‐AlPcs, but were not successful, including pentafluorophenoxy‐AlPc, 4‐nitrophenoxy‐AlPc and benzoate‐AlPc.

The resulting phenoxy‐AlPcs structures were confirmed by mass spectrometry and H^1^‐NMR in DMSO‐d_6_ (Figures S2–S6, Supporting Information). The protons closest to the oxygen on the phenoxy groups experienced a significant upfield shift (drop in ppm) in H^1^‐NMR signal upon coupling of the oxygen to the aluminum metal center of the Pc (Figures S2–S6, Supporting Information). Similar shifts have previously been reported for protons near oxygen in axially substituted silicon and tin Pcs.^[^ ^18^, ^58^ ^]^ Prior to device integration all materials were purified by train sublimation. The sublimation temperature of all phenoxy‐AlPcs were approximately 350 °C, which is significantly lower than the typical sublimation temperature for Cl‐AlPc at 410 °C, suggesting weaker intermolecular interactions that enable lower sublimation temperatures. The R‐AlPc molecules were characterized by both solution and solid‐state UV–Vis (Figure S7, Supporting Information). Characteristic peaks at 677–683 nm corresponded to the Q‐band of the Pcs, with the largest redshift observed for 345F‐AlPc and largest blue shift for oCr‐AlPc and pCr‐AlPc. In all cases, we observed a broadening and red shifting of the absorption out to 770 nm for the solid‐state samples, which are typical of solid‐state interactions. Minimal changes in optical bandgap due to phenoxylation is consistent with previously synthesized phenoxylated R‐BsubPcs,^[^ ^26^, ^53^, ^54^ ^]^ R_2_‐SiPcs^[^ ^38^ ^]^ and R‐AlPcs.^[^ ^55^ ^]^ The R‐AlPc molecules experienced limited solubility in most solvents, which are common for Pcs, limiting single crystal growth from solution. Sublimation also did not lead to single‐crystal formation. Regardless, we found that phenoxylation was an effective way to tune the molecular properties of R‐AlPc.

### 2.2 R‐AlPc Based Organic Thin‐Film Transistors

**Figure 2:**
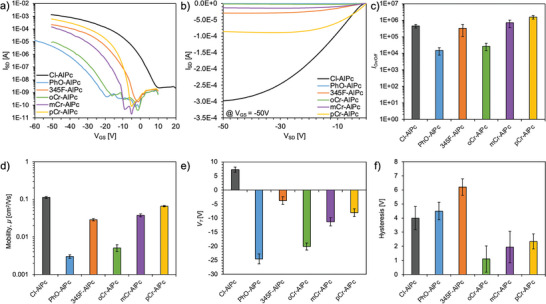
Baseline performance of R‐AlPc OTFTs, including the a) transfer and b) output curves of a representative device at a gate voltage of ‐50 V. The c) *I*_On/Off_, d) *µ*_h_, e) *V*_T_, and f) *H* were determined from the average of a minimum of 10 devices.

R‐AlPc compounds were incorporated into bottom‐gate bottom‐contact (BGBC) OTFTs as the semiconducting layer and compared to baseline Cl‐AlPc after testing in air. Device output and transfer curves were obtained and used to extract the transconductance (*g*_m_), saturation hole mobility (*µ*_h_), on–off current ratio (*I*_On/Off_), threshold voltage (*V*_T_) and hysteresis (*H*). All transfer and output curves can be found in Figures S8 and S9 (Supporting Information). As demonstrated in **Figure** **2**, Cl‐AlPc maintained the highest performance among all R‐AlPc compounds when integrated into OTFTs, with the greatest *µ*_h_, *g*_m_, and *I*_On_. However, pCr‐AlPc, mCr‐AlPc and 345F‐AlPc showed a comparable *µ*_h_ within the same order of magnitude as Cl‐AlPc, and comparable if not superior *V*_T_ and *I*_On/Off_. Additionally, the cresol‐substituted compounds (pCr‐AlPc, mCr‐AlPc, oCr‐AlPc) also experienced the lowest *H* of all R‐AlPc.

**Figure 3:**
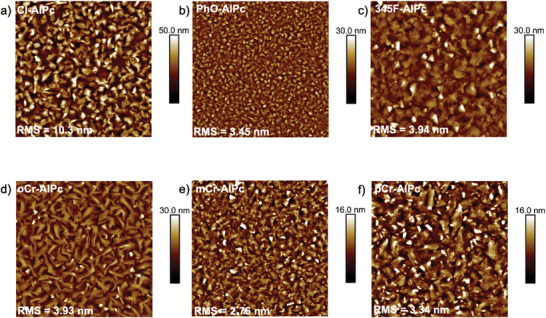
AFM images of a) Cl‐AlPc, b) PhO‐AlPc, c) 345F‐AlPc, d) oCr‐AlPc, e) mCr‐AlPc, and f) pCr‐AlP. All images are 2.5 µm × 2.5 µm, with a scale bar of 500 nm.

MPcs are known to crystallize in thin films such that small molecular changes, like the location of a methyl group on the axial phenoxy ring, can lead to widely different film morphologies and resulting OTFT performance.^[^ ^59^ ^]^ In this study, the lowest‐performing materials in devices were PhO‐AlPc and oCr‐AlPc, which both yielded a *µ*_h_ an order of magnitude lower than Cl‐AlPc and a large *V*_T_. A possible explanation for the poor performance of these substituted compounds lies in the molecular packing of these materials in thin‐films, which has an effect on the surface morphology, and subsequent charge transfer through the film.^[^ ^11^, ^13^ ^]^ Toward this end, the surface morphology of each R‐AlPc film was investigated by AFM (**Figure** **3**). The thin‐films of 345F‐AlPc, mCr‐AlPc and pCr‐AlPc all had similar morphologies, with highly crystalline domains and a similar mean surface roughness of 3–4 nm. Additionally, the shape and size of the grains are similar to that of the Cl‐AlPc film, forming larger, more plate‐like grains consistent with those previously reported for Cl‐AlPc.^[^ ^60^ ^]^ However, the PhO‐AlPc film shows substantially smaller crystal domains, while the oCr‐AlPc film shows much larger, irregularly shaped crystal structures compared to Cl‐AlPc. Both PhO‐AlPc and oCr‐AlPc film morphologies could account for the reduced charge transport efficiency observed from OTFTs derived from these semiconductors (Figure 2). The smaller crystal domains in the PhO‐AlPc film have a smaller area of tightly packed and aligned molecules with more grain boundaries throughout the film, which would have a higher impact on the resistance to charge transport.^[^ ^61^, ^62^ ^]^ The highly disordered grains observed in the oCr‐AlPc film are randomly oriented and have larger grain boundaries, which would also negatively impact charge transport. As demonstrated by the Cl‐AlPc, 345F‐AlPc, pCr‐AlPc and mCr‐AlPc films, highly crystalline domains of a moderate size strike a good balance, with high‐quality transport pathways available with fewer large grain boundaries.

To further compare the crystallinity and orientation, GIWAXS was performed on each of the films (Figure S10, Supporting Information), however since single crystal data could not be obtained for the R‐AlPc compounds, the resulting scattering could not be attributed to the specific packing of the molecules. From Figure S10 (Supporting Information), the highest performing devices appeared to have the broadest scattering signals and distribution of molecular orientations (Figure S23, Supporting Information). These films still demonstrate a high overall crystallinity by XRD (Figure S14, Supporting Information) and AFM (Figure 3) but are likely less uniformly aligned. However, their favorable grain structures and high crystallinity within those individual grains is likely driving the higher performance of these films. The scattering pattern observed for oCr‐AlPc was sharp with many high intensity reflections, indicating highly ordered crystalline domains with a strong preferential orientation to the substrate at a *χ* of 75° (Figure S23, Supporting Information), although the exact orientation of the Pc cannot be concluded due to the lack of single‐crystal XRD data. While the crystallinity of this film was high with a single preferential orientation, which is generally associated with improved device performance, this orientation could be less conducive to efficient charge transport in conjunction with the larger grain boundaries and irregular grain structures observed by AFM. Overall, the performance of phenoxy‐AlPc derivatives in OTFTs, paired with their film characterization, demonstrates that they can successfully act as semiconductors, and that phenoxylation can be used both to impart functionality to the Pc and provide a handle for crystal engineering and device optimization through molecular design.

### 2.3 Exposure of R‐AlPc Molecules to Cannabinoids

**Figure 4:**
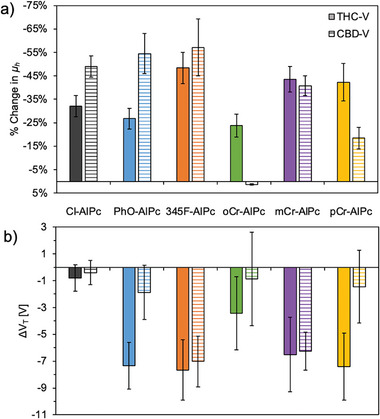
OTFT Performance before and after exposure to 300 ppm THC vapor and 300 ppm CBD vapor for 90 s. The change in a) *µ*_h_ and b) *V*_T_, were determined from the average of a minimum of 10 devices, where the x‐axis legend is the same for both (a,b).

To assess the performance of the R‐AlPc materials as a cannabinoid sensing layer, they were integrated into OTFT‐based sensors. The R‐AlPc OTFTs were characterized before and after exposure to 300 ppm THC vapor, 300 ppm CBD vapor (**Figures** **4** and S11, Supporting Information), 20 × 10^−6^ m THC solution and 20 × 10^−6^ m CBD solution (Figure S12, Supporting Information) to determine how the device performance changed in response to cannabinoid exposure. A similar sensor response, in terms of trends, between the R‐AlPcs and THC and CBD solution and vapor was observed, thus for simplicity this section will focus on the discussion of THC and CBD vapor. Overall, compared to Cl‐AlPc, devices made with 345F‐AlPc, mCr‐AlPc and pCr‐AlPc experienced a greater change in *µ*_h_, *g*_m_ and *I*_On/Off_, while devices made with all phenoxy‐AlPcs experienced larger shifts in *V*_T_ compared Cl‐AlPc (Figure 4). The performance of these materials surpasses those previously reported using Cl‐AlPc,^[^ ^12^ ^]^ but does not quite reach the sensitivity achieved using ZnPc as the sensing layer.^[^ ^13^ ^]^ Other previously reported MPc cannabinoid sensors have reached similar sensitivities, but have required the addition of a sensitizing probe to the surface to achieve these results.^[^ ^9^, ^10^, ^11^ ^]^

Additionally, some phenoxy‐AlPcs experienced selective responses to one cannabinoid over the other. However, the reason for this response is ultimately unknown. A selective response to THC and CBD has previously been reported for Cl‐AlPc and other MPcs in OTFTs,^[^ ^9^, ^10^, ^11^, ^12^ ^]^ and their interactions in solution have been investigated, suggesting strong coordination between the nitrogen ring and central metal of the Pc with the conjugated ring of the cannabinoids, causing changes in their electrical properties.^[^ ^10^ ^]^ It is unclear if these interactions in solution are reproduced in a thin‐film. The selectivity observed with the phenoxy‐AlPcs could be due to a combination of similar electrochemical interactions and the interactions of THC and CBD with the different crystal structures and morphologies of the phenoxy‐AlPcs. The absence of single crystal data for each of the phenoxy‐AlPcs makes it difficult to draw conclusions, but these results indicate that the choice of phenoxy group on the AlPc could be used to provide a more sensitive cannabinoid sensing layer in OTFT based sensors.

**Figure 5:**
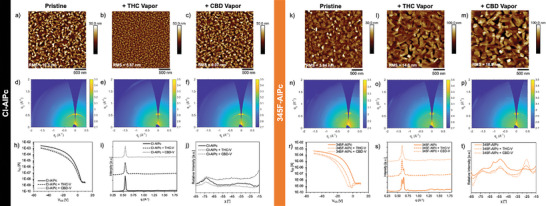
Film morphology and device performance of a–j) Cl‐AlPc and k–t) 345F‐AlPc films in response to 300 ppm THC vapor or CBD vapor. a–c,k–m) 2.5 µm × 2.5 µm AFM images. d–f,n–p) 2D GIWAXS spectra while i,s) are the corresponding diffraction patterns, and j) and t) are the linecut profiles with respect to *χ* for a *q* range of 0.5–0.65. h,r) OTFT transfer curves.

As previously observed, THC and CBD can coordinate strongly with R‐AlPc molecules, changing the Pcs electronic properties, and therefore the substituted phenoxy group could improve these interactions.^[^ ^10^ ^]^ Additionally, THC and CBD can induce structural changes in thin films, also leading to reductions in *µ*_h_, *I*_On/Off_, *V*_T_, and changes in subthreshold slope and defect density.^[^ ^11^, ^12^, ^13^ ^]^ **Figure** **5** represents the AFM, GIWAXS and OTFT transfer curves for 345F‐AlPc and Cl‐AlPc before and after exposure to THC or CBD vapor to investigate the effect of phenoxy functionalization of AlPc. The AFM images of 345F‐AlPc films show significant reorganization or recrystallization after exposure to THC or CBD, leading to larger crystals with wider grain boundaries and increased surface roughness from 3.9 to 14.8 nm and 18.9 nm, respectively. In comparison, the same exposure led to a reduction in grain size for Cl‐AlPc films and a drop in surface roughness from 10.3 to 5.6 nm and 9.06 nm for THC and CBD, respectively (Figure 5). Changes in grain size and film morphology can affect the number and quality of charge transport pathways available, altering the defect density and causing a reduction in performance as a result of structural interactions between THC or CBD with Pc thin films.^[^ ^11^ ^]^ An increase in grain size, as seen with 345F‐AlPc, can lead to a decrease in defect density due to the larger crystal structures (Figure S11, Supporting Information), but a simultaneous decrease in the quality of charge transport pathways due to the larger grain boundaries and more irregular grains, leading to the observed overall reduction in *µ_h_* and *V_T_*.^[^ ^38^, ^61^, ^63^ ^]^ A similar trend was observed with the other phenoxy‐AlPc films (Figures S15 and S16, Supporting Information). The orientation of the molecules within these grains may also help explain the reduced performance.

The 2D scattering patterns and the azimuthally integrated GIWAXS patterns of Cl‐AlPc thin‐films displayed in Figure 5 exhibit no large structural changes after THC or CBD exposure. However, exposure to THC and CBD vapor resulted in a change in molecular order of the Cl‐AlPc film with a slight increase in film crystallinity evident by the rounded reflection at *q* = 0.56 Å^−1^, compared to the more arched shaped of the same reflection pre‐exposure to cannabinoids. These results are consistent with previous reports for Cl‐AlPc films upon exposure to THC and CBD (liquid solution or vapor).^[^ ^12^ ^]^ Conversely, 345F‐AlPc films experience significant microstructural changes while maintaining approximately the same degree of molecular order and crystallinity. The initial thin films display high intensity reflections at *q* = 0.61 Å^−1^ and *q* = 0.65 Å^−1^ along the *q_z_* axis. When exposed to THC or CBD, a change in the ratio of these two peaks suggests a reorganization in the thin‐film. A change in the average molecular orientation was also detected from primarily ≈52° to a more mixed orientation with ≈25°, determined by linecuts with respect to χ (Figure 5). This means there could be a mix of molecules oriented more face‐on than edge‐on to the substrate upon exposure to cannabinoids, which is associated with reduced mobility and lower *I*_On_.^[^ ^13^, ^38^ ^]^ This can help explain the observed reduced *µ*_h_ with the larger crystallite size seen by AFM, which is generally associated with improved *µ*_h_. Similar relative changes in peak intensities from the (100) plane at *q* = ≈0.50 Å^−1^ and (10‐2) plane at *q* = 0.68 Å^−1^ of β‐ZnPc films have been reported after exposure to THC vapor in conjunction with a shift in the average molecular orientation from ≈45° to ≈74° for β‐ZnPc molecules relative to the substrate.^[^ ^13^ ^]^ The more face‐on oriented films had a lower *I*_On/Off_, and lower *µ*_h_.^[^ ^13^ ^]^ While no single crystal data are available for the phenoxy‐AlPc derivatives, a similar shift in 345F‐AlPc molecular orientation is likely taking place with exposure to THC and CBD due to the similar changes detected by GIWAXS. In fact, all other phenoxy‐AlPc films experienced similar shifts in their film morphology (Figures S13 and S15–S17, Supporting Information), with pronounced grain restructuring and crystal reorganization similar to that of 345F‐AlPc. The integrated GIWAXS patterns demonstrate that the phenoxy substitutions promote more reorganization of R‐AlPc films upon THC vapor exposure that is not observed with Cl‐AlPc. By GIWAXS (Figure S10, Supporting Information) and XRD (Figure S14, Supporting Information), the d‐spacing of the phenoxylated‐AlPc films are smaller than Cl‐AlPc, suggesting smaller intermolecular distances. However, as evidenced by lower sublimation temperature, the phenoxy groups are likely disrupting the strength of the intermolecular interactions, corroborating the higher tendency for the phenoxy‐AlPc films to reorganize upon exposure to cannabinoids.

The R‐AlPc films were also exposed to a 20 × 10^−6^ m liquid THC solution, and 20 × 10^−6^ m CBD solution, where similar results were observed (Figures S18–S23, Supporting Information). Significant changes in the thin‐film morphology were observed for the phenoxy‐AlPc films, resulting in the larger changes observed in their device performance. Again, 345F‐AlPc experienced significant surface morphology changes in response to THC and CBD vapor resulting in an increase in grain size and surface roughness. This surface reorganization was less significant upon exposure to THC and CBD solutions, suggesting the solvent interferes with the surface interactions. These results demonstrate selective effects of THC relative to CBD which can facilitate ratiometric cannabinoid analysis,^[^ ^9^ ^]^ and the improvement of OTFT sensitivity to both cannabinoids with the implementation of phenoxylated AlPc.

Finally, Raman spectroscopy was used to further probe the interactions between the R‐AlPc molecules and cannabinoids in a thin film (Figure S24, Supporting Information). Upon exposure, there appears to be shifting of the peaks in both location and intensity around the isoindole region of the spectra, at ≈1530–1540 cm^−1^.^[^ ^64^, ^65^ ^]^ Spectra of THC and CBD alone were collected to confirm that these shifts were not caused merely by the presence of the cannabinoids (Figure S25, Supporting Information). These results indicate a change in the environment around the R‐AlPc molecules as a result of interaction with THC/CBD in the thin film, suggesting the interactions between the MPc and the analyte are present in the solid state.

Phenoxylation of R‐AlPc alters the crystal packing of R‐AlPc molecules, reducing the strength of the intermolecular interactions compared to Cl‐AlPc. As THC has been shown to coordinate with the nitrogen ring of AlPcs in previous work,^[^ ^10^ ^]^ the tight crystal structure is likely interrupted by the introduction of THC, causing more drastic shifts in film structure. Consequently, the addition of the phenoxy groups changed the response of AlPc to analytes and generated a more sensitive cannabinoid sensor through the introduction of new crystal structures. This demonstrates the utility of molecular engineering as a strategy to fine tune the thin‐film properties of Pcs for sensor optimization through the enhancement of Pc‐analyte interactions.

## 3 Conclusion

This work demonstrates the successful synthesis and integration of phenoxylated AlPc molecules in OTFT cannabinoid sensors for the first time. Through the introduction of new crystal structures and intermolecular interactions, the phenoxy‐AlPc molecules have shown an improved response to THC compared to Cl‐AlPc, while maintaining a comparable baseline device performance. The most sensitive of the substitutions, 345F‐AlPc, experienced drastic changes in grain size and film roughness, thin‐film reorganization and crystal orientation upon exposure to THC vapor that were not detected with Cl‐AlPc. These structural changes within the thin film drive the improved sensitivity to THC and highlight molecular engineering as a straightforward technique to tune and enhance analyte‐semiconductor interactions in OTFT sensors.

## 4 Experimental Section

### Materials

Cl‐AlPc (85% purity) was received from Abacipharm and used in chemical reactions as received. It was purified by train sublimation before use in OTFTs. Phenol, o‐cresol, p‐cresol, and 3,4,5‐trifluorophenol were supplied by TCI Chemicals, and m‐cresol was supplied by Sigma‐Aldrich. All were used as received. (Octyl)trichlorosilane (OTS, 97%) was purchased from TCI Chemicals and used as received. Chlorobenzene (99%) was purchased from Sigma‐Aldrich. DMSO‐d_6_ was supplied by Cambridge Isotope Laboratories Ltd. All other solvents used in device fabrication were HPLC grade and purchased from Fisher Scientific. Toronto Research Chemicals supplied the cannabinoid standards.

### Chemical Characterization

NMR spectrometry of all newly synthesized compounds was performed on a Bruker AVANCE III 400 NMR spectrometer. Mass spectrometry analysis was performed at the University of Toronto AIMS Mass Spectrometry Laboratory.

### Chemical Synthesis

A 100 mL round‐bottom flask was charged with 1 g of Cl‐AlPc (1.74 mmol) and a 10‐molar excess of the substituted phenol in 30 mL of chlorobenzene. The flask was stirred under nitrogen and refluxed at 125 °C for 18 h. Upon cooling, the solvent was evaporated and the solid was washed with 3 m KOH, filtered, and dried. The product was purified by train sublimation at 350– 380 °C, depending on the compound, to obtain a pure final product for use in device fabrication. The structure of all synthesized compounds was confirmed by NMR spectroscopy (Figures S2–S6, Supporting Information) and mass spectrometry (Figures S26–S30, Supporting Information). The details and results for each individual reaction can be found in Table S1 (Supporting Information).

### Thin‐Film Deposition

Fraunhofer IPMS supplied prefabricated devices to make BGBC transistors, each consisting of a 230 nm thermally grown SiO_2_ dielectric layer on an Si gate, and prepatterned source–drain electrodes with a *W/L* of 1000 (*W* = 10 mm, *L* = 10 µm). Substrates purchased from Ossila were used for AFM and XRD characterization, consisting of a 300 nm thermally grown SiO_2_ dielectric layer on Si. The substrates were completely rinsed with acetone to remove the photoresist, then washed with isopropanol and dried under a N_2_ gas stream. The substrates were treated with oxygen plasma for 15 min then briefly rinsed with water, isopropanol, and dried with N_2_. Once dry, they were immersed in a 1% v/v OTS solution in toluene for 1 h at 70 °C. The substrates were then rinsed with toluene and isopropanol, dried with N_2_, and placed in a vacuum oven at 70 °C for 1 h to remove trace solvent. The substrates were placed in an Angstrom EvoVac thermal evaporator at a pressure below 2 × 10^−6^ Torr and heated to 140 °C, and the temperature was stabilized and held for 1 h. Deposition of R‐AlPc was performed by sublimation at 0.2 Å s^−1^ until a thickness of 500 Å was reached. The substrates were cooled to room temperature before physical and electrical characterization.

### AFM Characterization

AFM measurements (2.5 µm × 2.5 µm) were performed with a Bruker Dimension Icon AFM with ScanAsyst‐Air tips and a scan rate of 0.69 Hz. Processing and editing of images were performed using NanoScope Analysis v.1.8 software.

### XRD Characterization

XRD measurements were performed using a Rigaku Ultima IV powder diffractometer with a Cu‐Kα (*λ* = 1.5418 Å) source with a scan range of 3° < 2*θ* < 18° at a rate of 0.5° min^−1^.

### GIWAXS Characterization

GIWAXS experiments were performed at the Canadian Light Source (CLS) in Saskatoon, Canada on the Brockhouse Diffraction Sector Undulator (BXDS IVU) beamline with a photon energy of 10 keV. The scattering data was collected on a Rayonix MX300 CCD detector (73.242 µm × 73.242 µm pixel size), placed 401.5 mm from the sample, at an angle of *θ* = 0.3°. All data were calibrated with silver behenate and poly(3‐hexylthiophene‐2,5‐diyl) standards and analyzed with the GIXSGUI software package in MATLAB, where both polarization and solid‐angle corrections were applied.

### Raman Characterization

Raman spectra were collected on a Renishaw inVia Raman microscope using a 532 nm laser with 2400 l/mm grating. The spectra for the R‐AlPc films were collected as a sum of 5 accumulations at 10% power, with 1 s exposure per measurement over a wavelength range of 550–1700 cm^−1^. The THC and CBD‐only spectra was performed on a thin film of pure cannabinoid oil on a glass slide, and were collected as a sum of 5 accumulations at 10% power with 5 s exposure per measurement over a wavelength range of 550–1700 cm^−1^. A baseline subtraction was performed to remove the baseline noise from the measurement.

### OTFT Characterization

Transistors were tested using a custom‐build auto tester machine and run using a Keithley 2614B SourceMeter and Labview software. The *V*_SD_ and *V*_GS_ were set and the *I*_SD_ was measured to obtain both output and transfer curves. Six transfer curves were taken for each transistor and the final three were averaged. From the transfer curve, the *V*_T_, *H*, and *µ*_h_ could be extracted, the latter using Equation 1.

(1) $$I_{\mathrm{SD}}=\frac{\mu C_{i}W}{2L}\;{\left(V_{\mathrm{GS}}-V_{T}\right)}^{2}$$

Once the baseline performance of all R‐AlPc compounds was assessed, new transistors were exposed to either a liquid THC solution (20 × 10^−6^ m) in hexanes, a liquid CBD solution (20 × 10^−6^ m) in hexanes, THC vapor (300 ppm concentration) or CBD vapor (300 ppm concentration). For the liquid solutions, 0.5 µL was dropcast on the transistor surface and allowed to dry for 2 min. For vapor exposure, the samples were vaporized into an 8 L bag using the Vapormed Volcano Medic, and the bag was slowly emptied over the devices in a chamber for 90 s. New output and transfer curves were then obtained for all exposed devices, allowing for a comparison with the baseline values to assess the sensitivity of each R‐AlPc compound as a sensing layer for OTFT cannabinoid sensors.

## Conflict of Interest

The authors declare no conflict of interest.

## Supporting information

Supporting Information

## Data Availability

The data that support the findings of this study are available from the corresponding author upon reasonable request.
