# Supplementary material for: Axial Phenoxylation of Aluminum Phthalocyanines for Improved Cannabinoid Sensitivity in OTFT Sensors
Source: Adv Sci (Weinh). 2024 Apr 19;11(27):2305515. doi: 10.1002/advs.202305515 (PMC11251552; doi:10.1002/advs.202305515)
Supplement: Supplementary file 1 — Supporting Information [file ADVS-11-2305515-s001.pdf]

ADVANCED SCIENCE
Open Access

Supporting Information

for *Adv. Sci.*, DOI 10.1002/advs.202305515

Axial Phenoxylation of Aluminum Phthalocyanines for Improved Cannabinoid Sensitivity in OTFT Sensors

Halynne R. Lamontagne, Rosemary R. Cranston, Zachary J. Comeau, Cory S. Harris, Adam J. Shuhendler and Benoît H. Lessard**

Supporting Information

Axial Phenoxylation of Aluminum Phthalocyanines for Improved Cannabinoid Sensitivity in OTFT Sensors

Halynne R. Lamontagne[1,2], Rosemary R. Cranston[1], Zachary J. Comeau[3], Cory S. Harris[4], Adam J. Shuhendler[2,4,5*] and Benoit H. Lessard[1,6*]

[1]*Department of Chemical and Biological Engineering, University of Ottawa, 161 Louis Pasteur, Ottawa, ON, Canada, K1N 6N5*

[2]*Department of Chemistry and Biomolecular Sciences, University of Ottawa, 150 Louis Pasteur, Ottawa, ON, Canada, K1N 6N5*

[3]*Advanced Electronics and Devices, National Research Council Canada, 1200 Montreal Rd, Ottawa, ON, Canada, K1A 0R6*

[4]*Department of Biology, University of Ottawa, 30 Marie Curie, Ottawa, ON, Canada, K1N 6N5*

[5]*University of Ottawa Heart Institute, 40 Ruskin St, Ottawa, ON, Canada, K1Y 4W7*

[6]*School of Electrical Engineering and Computer Science, University of Ottawa, 800 King Edward Ave, Ottawa, ON, Canada, K1N 6N5*

*Co-Corresponding Authors: Adam.Shuhendler@uottawa.ca (AJS) Benoit.Lessard@uottawa.ca (BHL)

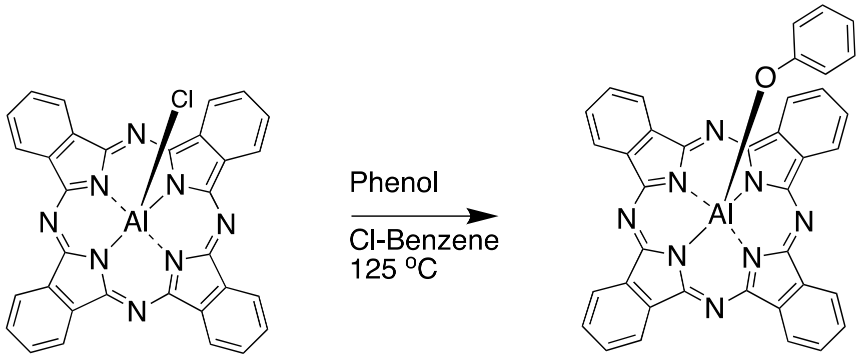

Figure S1. Phenoxylation reaction of Cl-AlPc.

Table S1. Synthesis results for R-AlPc substituents.

R-Group	Reaction Yield After Sublimation	Expected Mass	Confirmed Mass	Confirmed Structure by 1H-NMR
Phenol (2)	16%	632.17	632.17	Yes
3,4,5-Trifluorophenol (3)	38%	686.14	686.14	Yes
o-Cresol (4)	36%	646.18	646.18	Yes
m-Cresol (5)	28%	646.18	646.18	Yes
p-Cresol (6)	13%	646.18	646.18	Yes

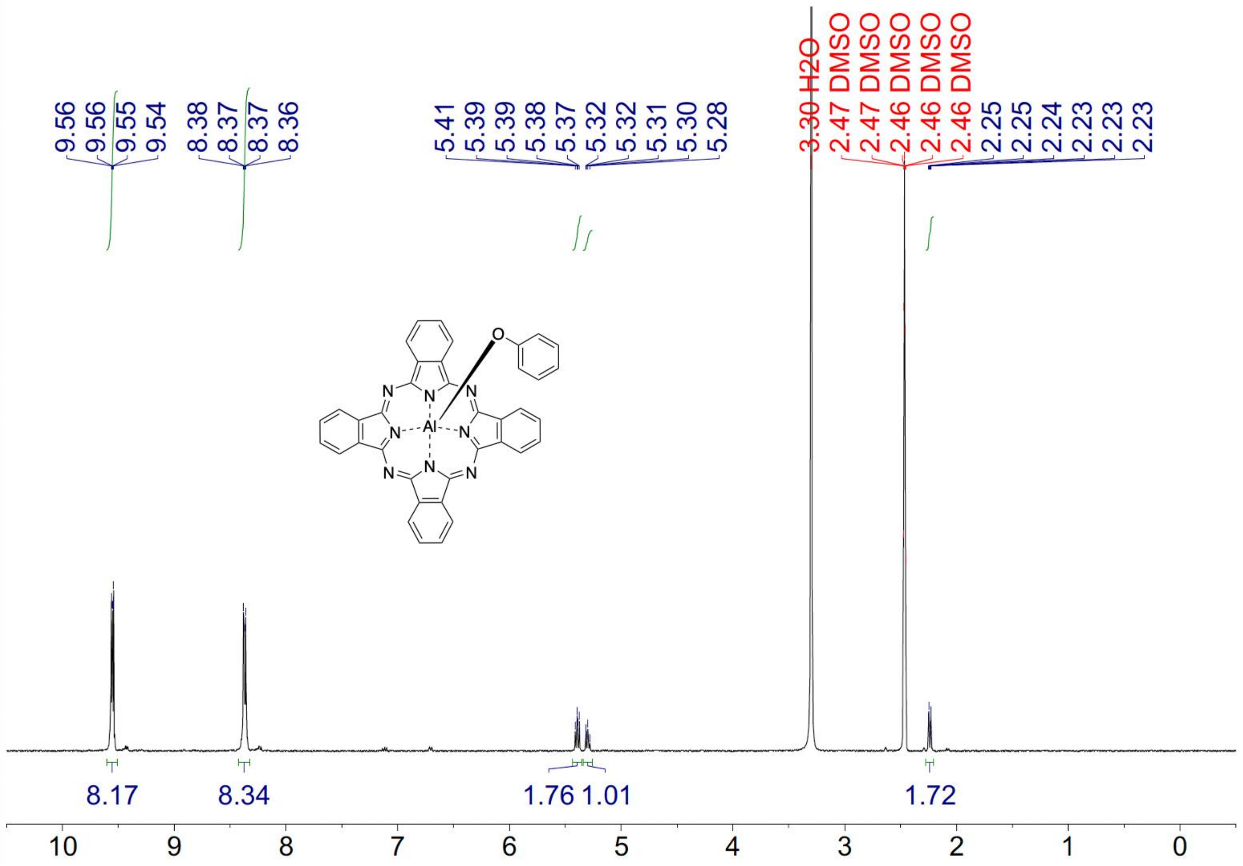

Figure S2. 1H-NMR of PhO-AlPc (2). (400 MHz; DMSO-d_6): δ 9.55 (m, 8H), 8.37 (m, 8H), 5.39 (m, 2H), 5.30 (m, 1H), 2.24 (m, 2H)

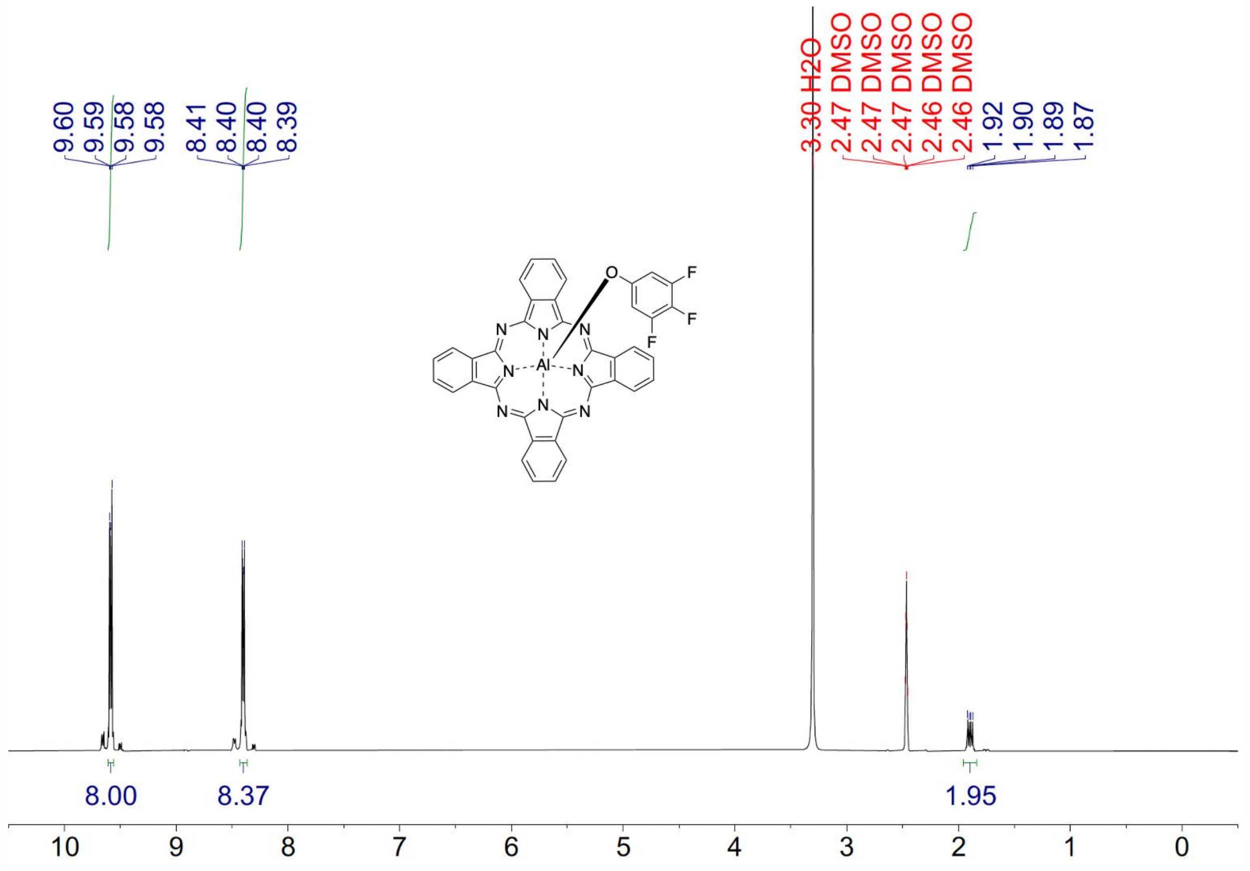

Figure S3. 1H-NMR of 345F_3-AlPc (3). (400 MHz; DMSO-d_6): δ 9.62 (m, 8H), 8.43 (m, 8H), 1.93 (m, 2H)

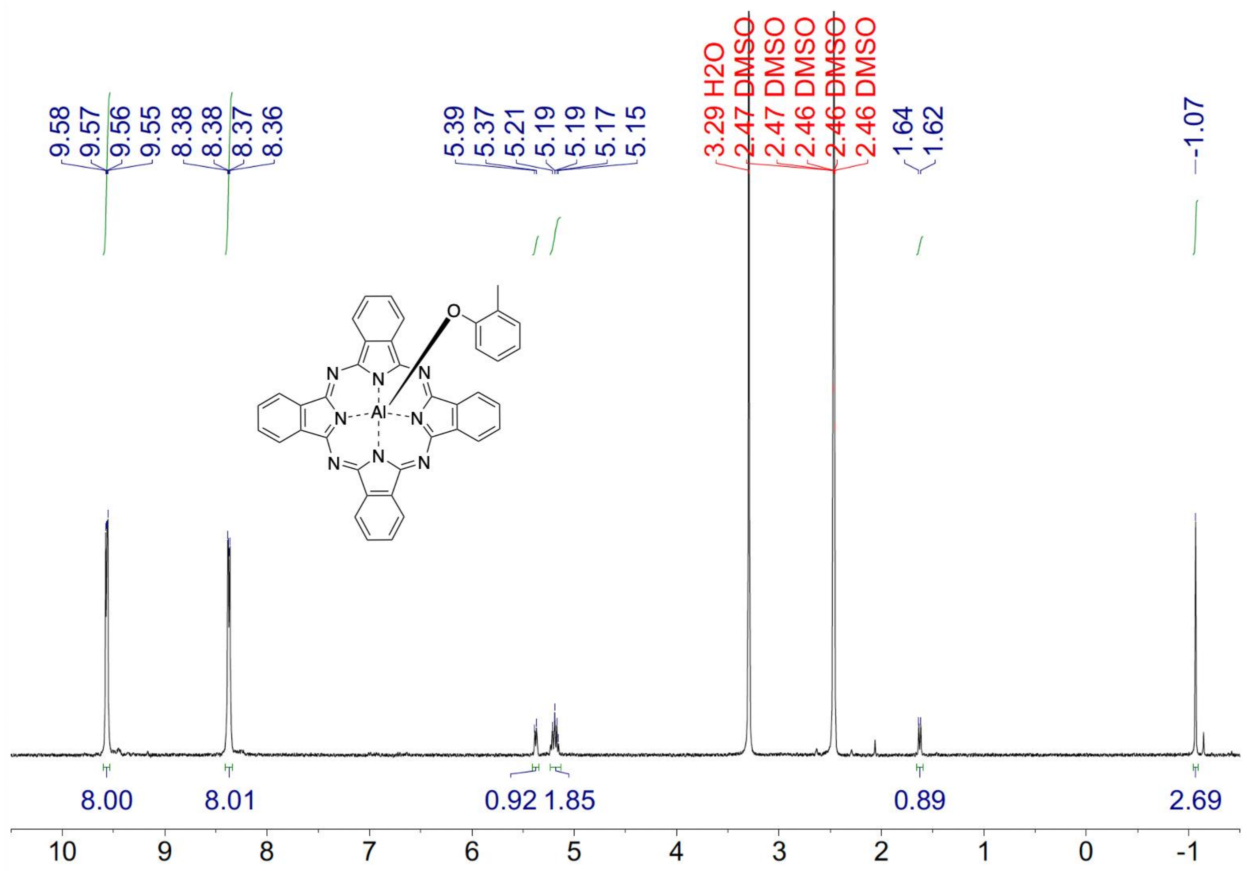

Figure S4. 1H-NMR of oCr-AlPc (4). (400 MHz; DMSO-d_6): δ 9.57 (m, 8H), 8.37 (m, 8H), 5.38 (m, 1H), 5.19 (m, 2H), 1.63 (d, *J* = 7.9 Hz, 1H), -1.07 (s, 3H).

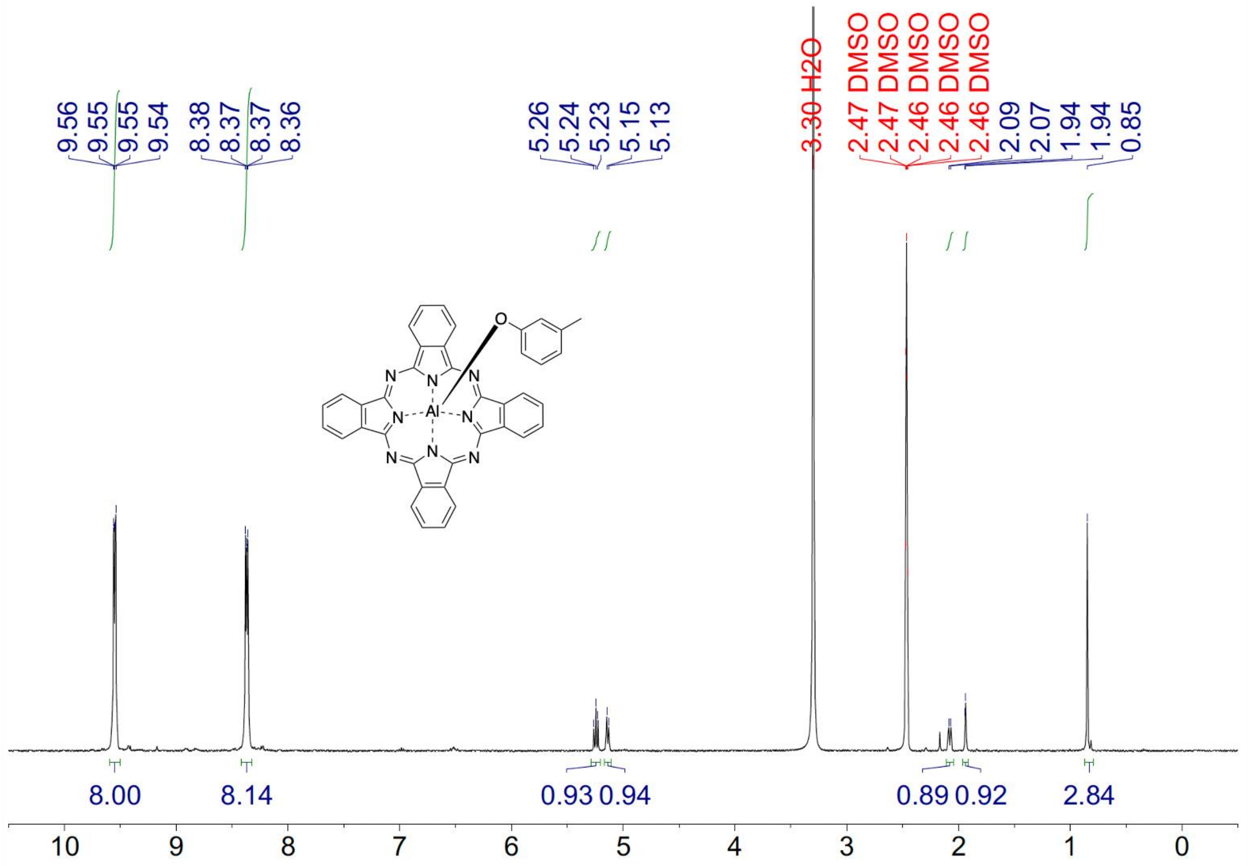

Figure S5. 1H-NMR of mCr-AlPc (5). (400 MHz; DMSO-d_6): δ 9.55 (m, 8H), 8.37 (m, 8H), 5.24 (t, *J* = 7.7 Hz, 1H), 5.14 (d, *J* = 7.3 Hz, 1H), 2.08 (d, *J* = 8.0 Hz, 1H), 1.94 (s, 1H), 0.85 (s, 3H).

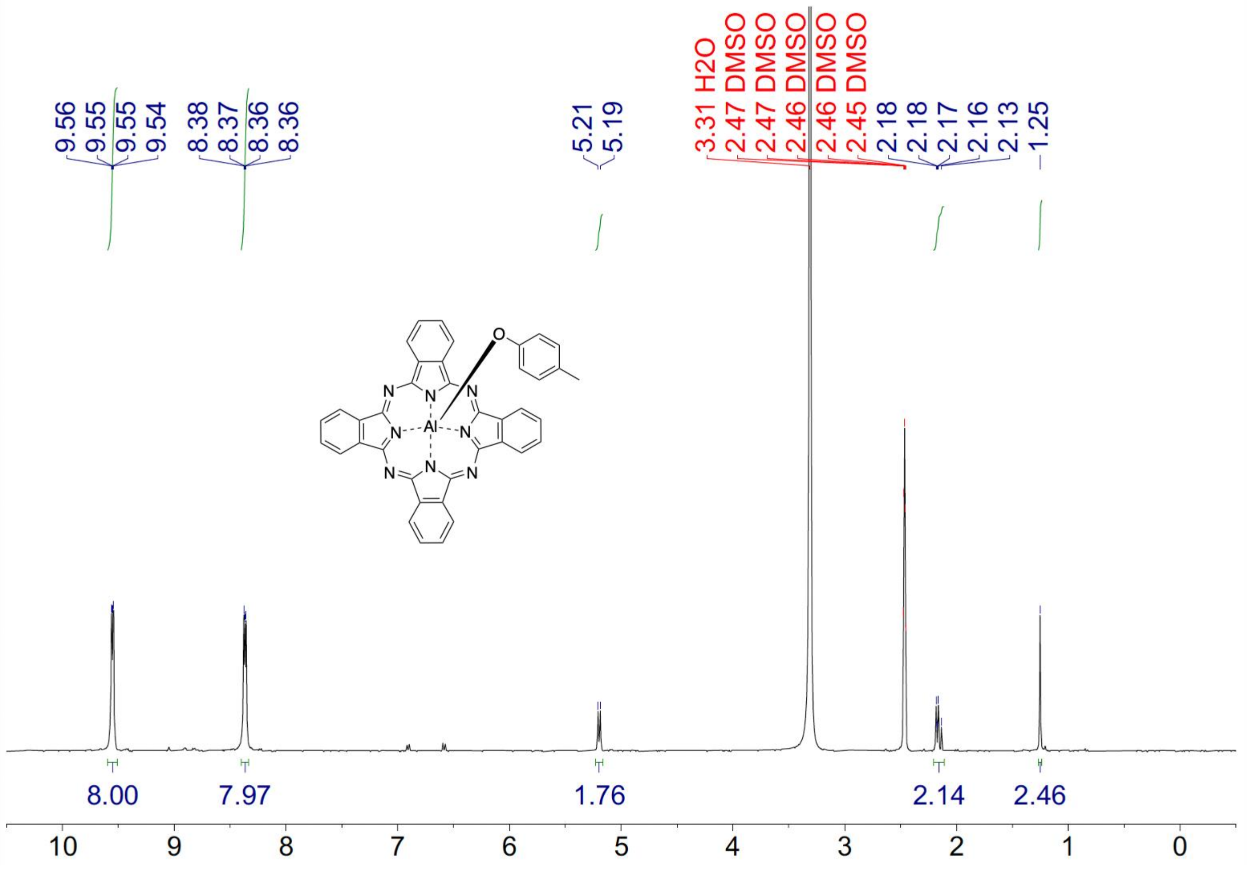

Figure S6. 1H-NMR of pCr-AlPc (6). (400 MHz; DMSO-d_6): δ 9.56 (m, 8H), 8.37 (m, 8H), 5.20 (d, *J* = 8.1 Hz, 2H), 2.16 (d, *J* = 7.3 Hz, 1H), 2.17 (m, 2H), 1.94 (s, 1H), 1.25 (s, 3H).

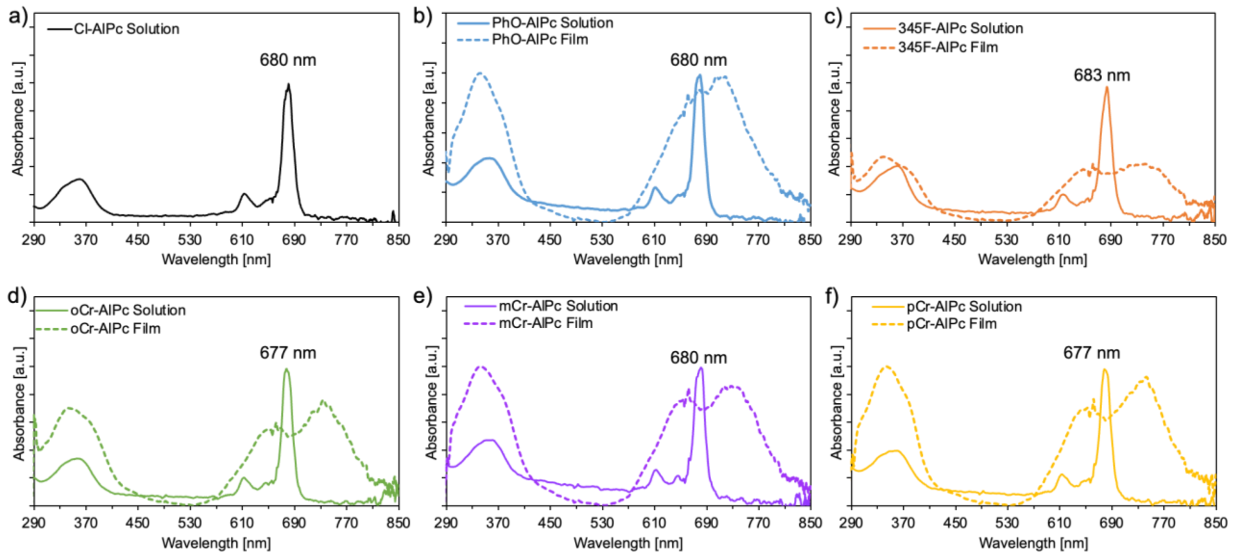

Figure S7. UV-Vis of substituted aluminum phthalocyanine molecules, including (a) Cl-AlPc, (b) PhO-AlPc, (c) 345F-AlPc, (d) oCr-AlPc, (e) mCr-AlPc, and (f) pCr-AlPc. Solution samples are indicated by a solid line, and solid-state samples are indicated by a dashed line.

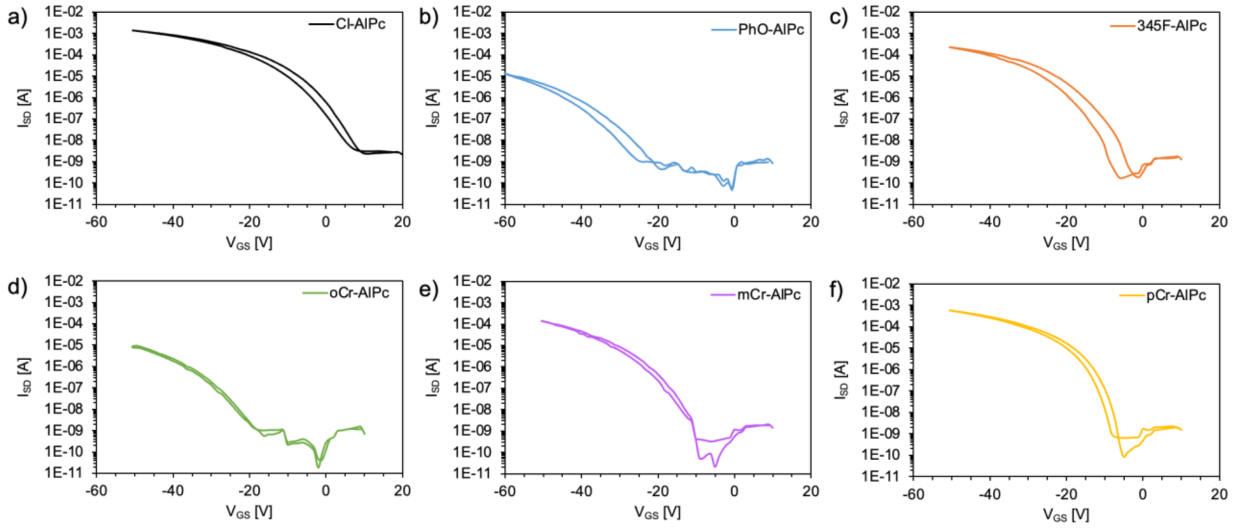

Figure S8. Forward and reverse transfer curves of R-AlPc OTFTs, (a) Cl-AlPc, (b) PhO-AlPc, (c) 345F-AlPc, (d) oCr-AlPc, (e) mCr-AlPc, and (f) pCr-AlPc.

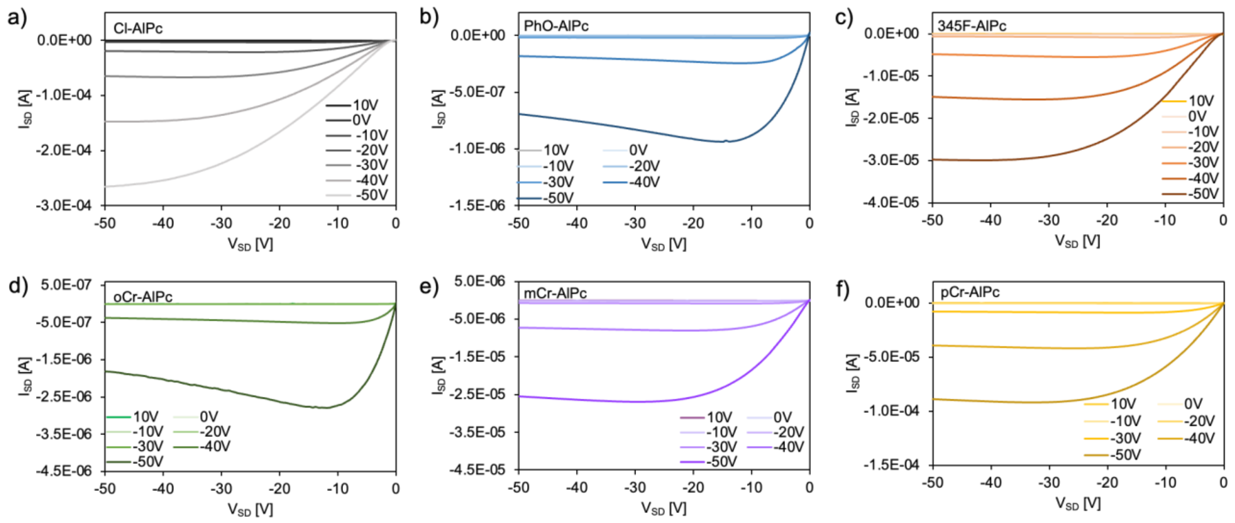

Figure S9. Output curves R-AlPc OTFTs, (a) Cl-AlPc, (b) PhO-AlPc, (c) 345F-AlPc, (d) oCr-AlPc, (e) mCr-AlPc, and (f) pCr-AlPc.

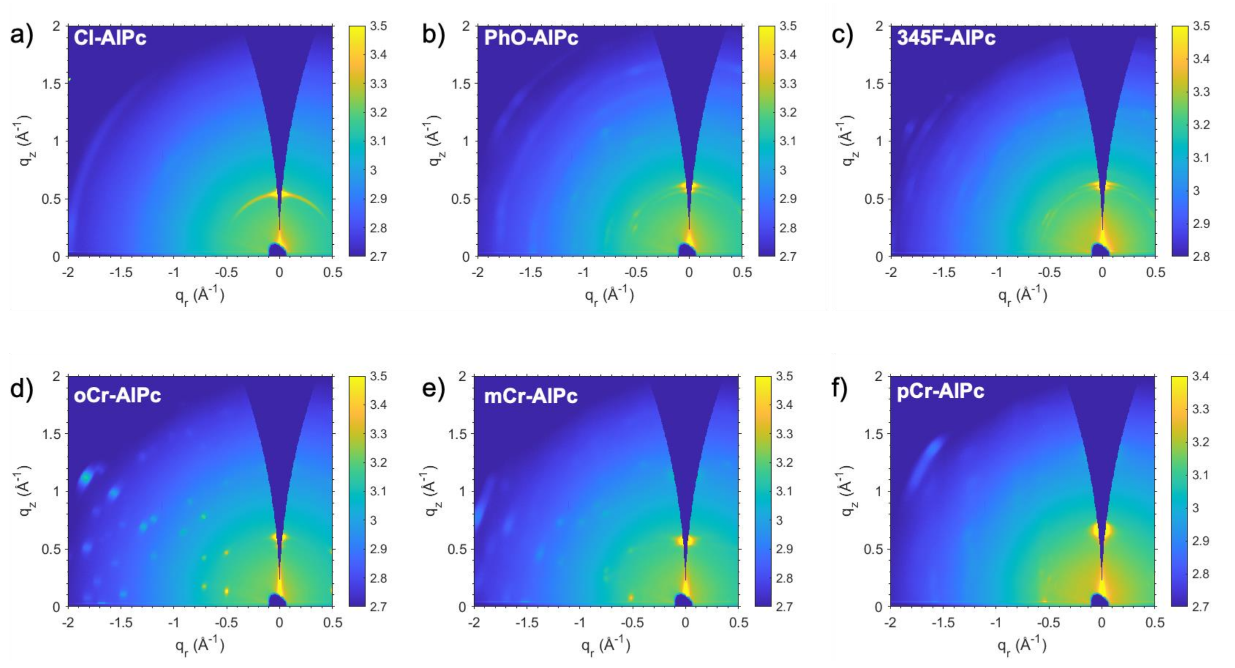

Figure S10. 2D GIWAXS spectra of (a) Cl-AlPc, (b) PhO-AlPc, (c) 345F-AlPc, (d) oCr-AlPc, (e) mCr-AlPc, and (f) pCr-AlPc films.

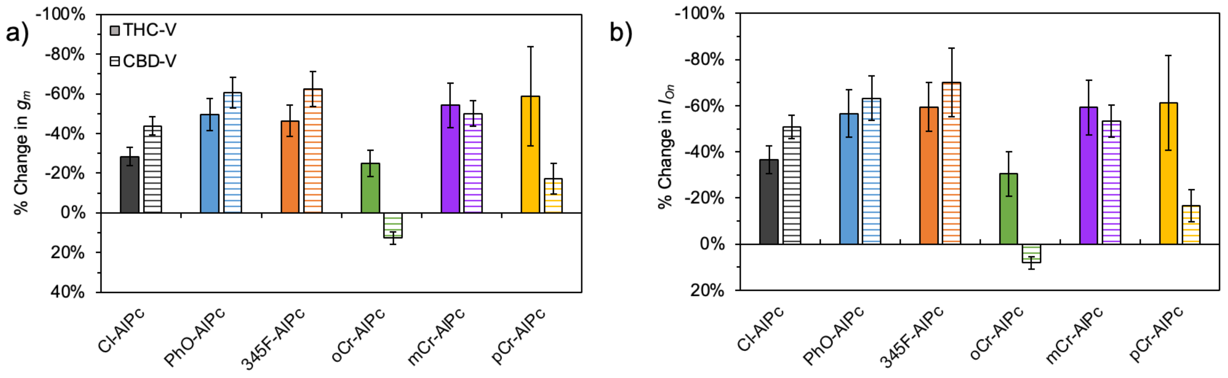

Figure S11. OTFT performance before and after exposure to 300 ppm THC vapor (THC-V) or 300 ppm CBD vapor (CBD-V) for 90 seconds. The change in (a) g_m, and (b) I_{On} were determined from the average of a minimum of 10 devices.

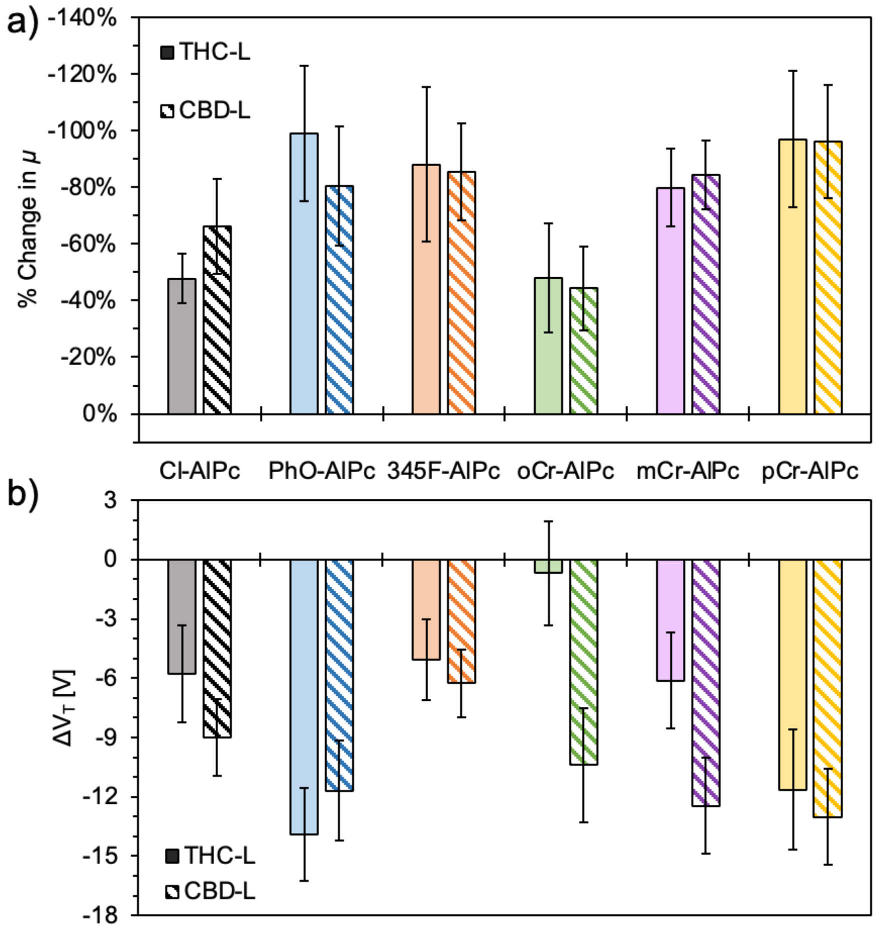

Figure S12. OTFT performance before and after exposure to 20 μM THC (THC-L) or CBD (CBD-L) solution in hexanes. The change in (a) μ_h and (b) V_T were determined from the average of a minimum of 10 devices, where the x-axis legend is the same for both (a) and (b).

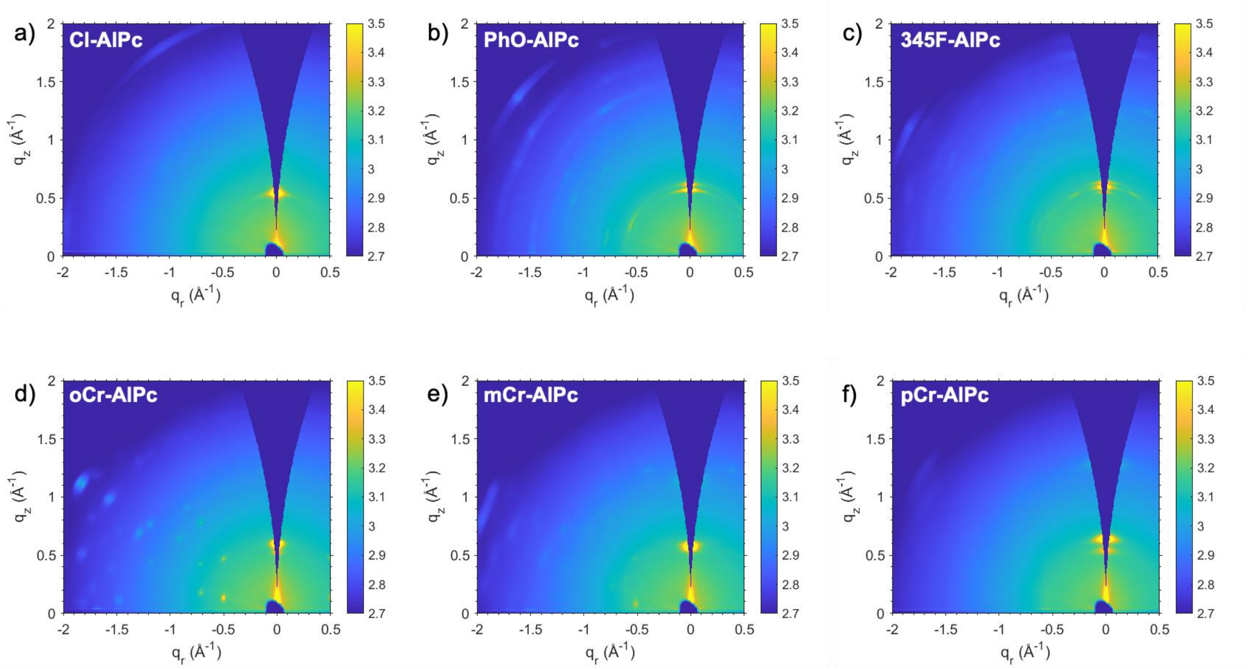

Figure S13. 2D GIWAXS spectra of (a) Cl-AlPc, (b) PhO-AlPc, (c) 345F-AlPc, (d) oCr-AlPc, (e) mCr-AlPc, and (f) pCr-AlPc films exposed to 300 ppm THC vapor for 90 seconds.

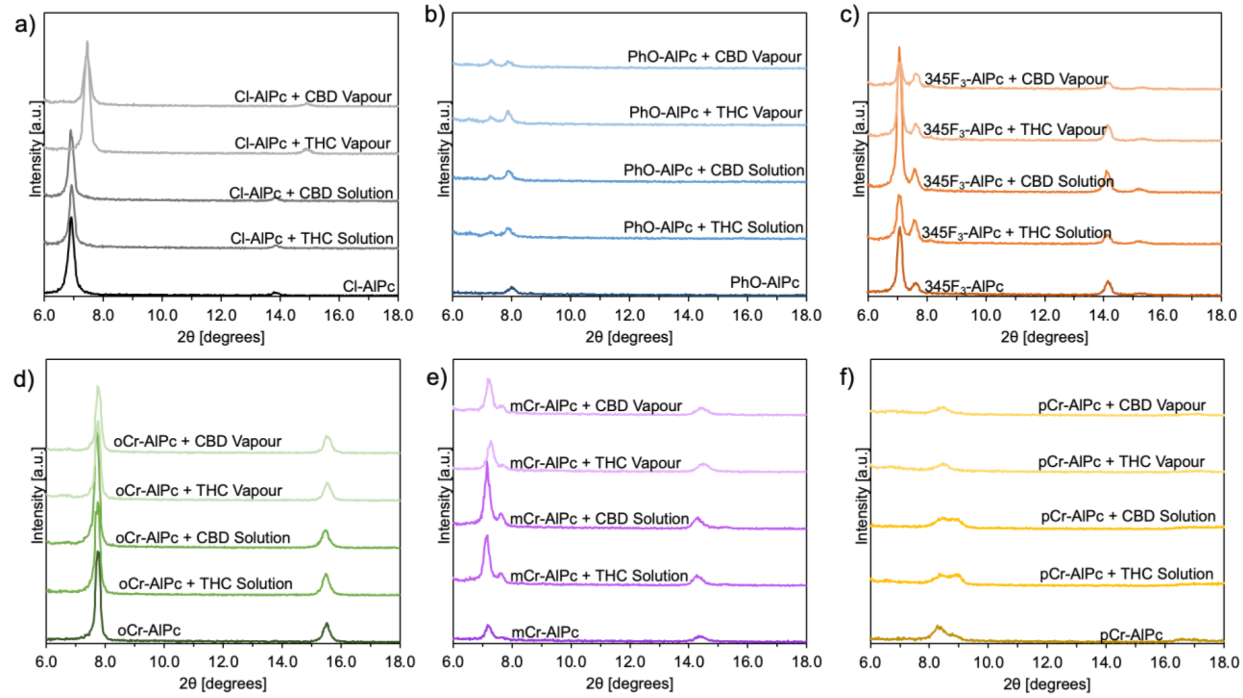

Figure S14. XRD spectra of (a) Cl-AlPc, (b) PhO-AlPc, (c) 345F-AlPc, (d) oCr-AlPc, (e) mCr-AlPc, and (f) pCr-AlPc thin films, before and after exposure to 20 μM of THC or CBD solution in hexanes, or 300 ppm THC or CBD vapor for 90 seconds.

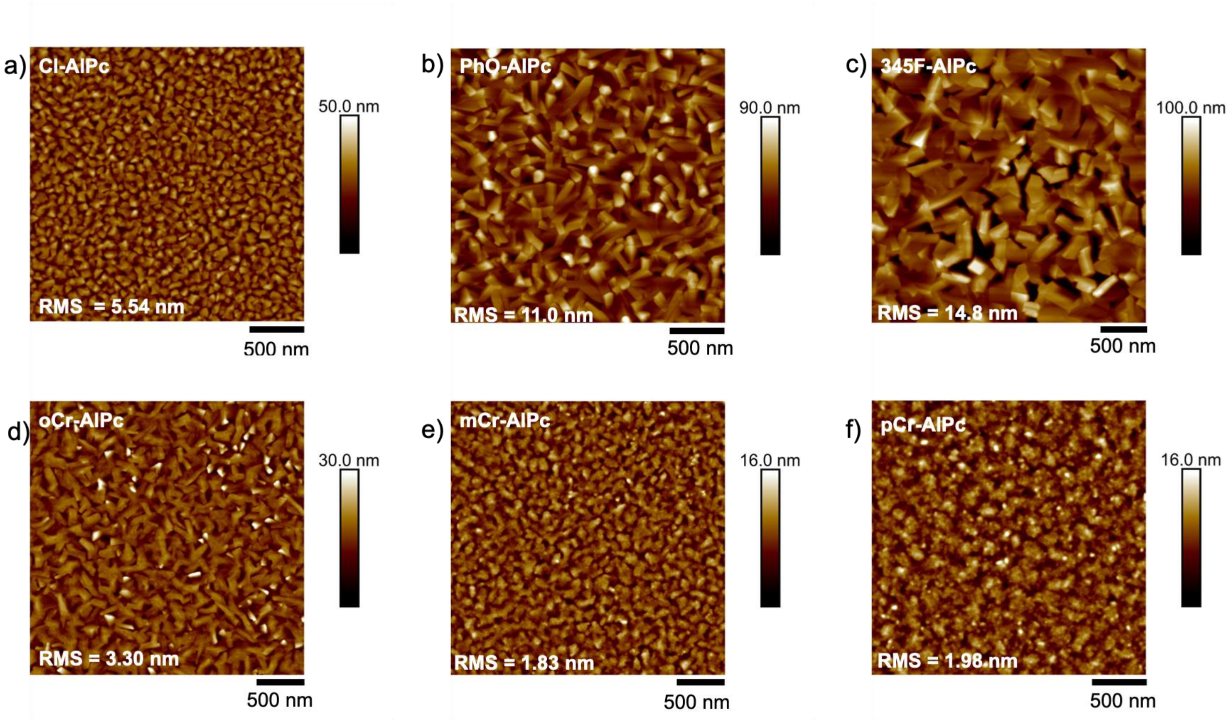

Figure S15. AFM images of (a) Cl-AlPc, (b) PhO-AlPc, (c) 345F_3-AlPc, (d) oCr-AlPc, (e) mCr-AlPc, and (f) pCr-AlPc, after exposure to 300 ppm THC vapor for 90 seconds. All images are 2.5 µm x 2.5 µm, with a scale bar of 500 nm.

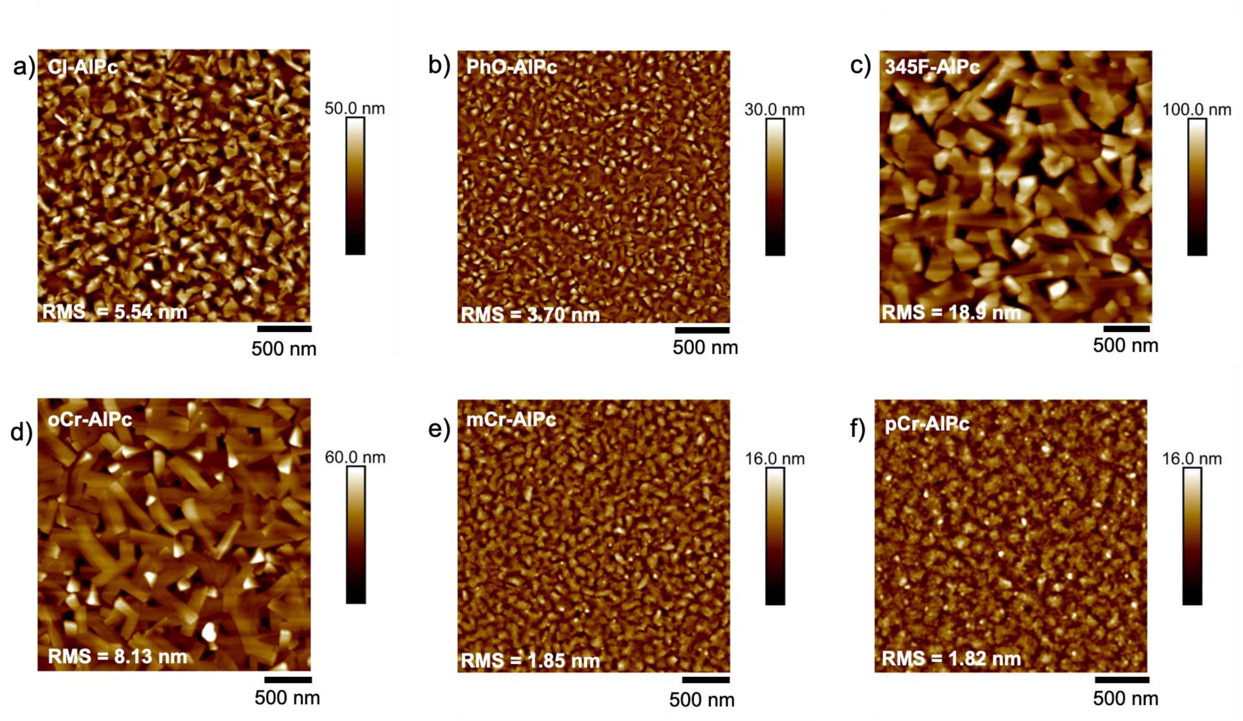

Figure S16. AFM images of (a) Cl-AlPc, (b) PhO-AlPc, (c) 345F_3-AlPc, (d) oCr-AlPc, (e) mCr-AlPc, and (f) pCr-AlPc, after exposure to 300 ppm CBD vapor for 90 seconds. All images are 2.5 μm x 2.5 μm, with a scale bar of 500 nm.

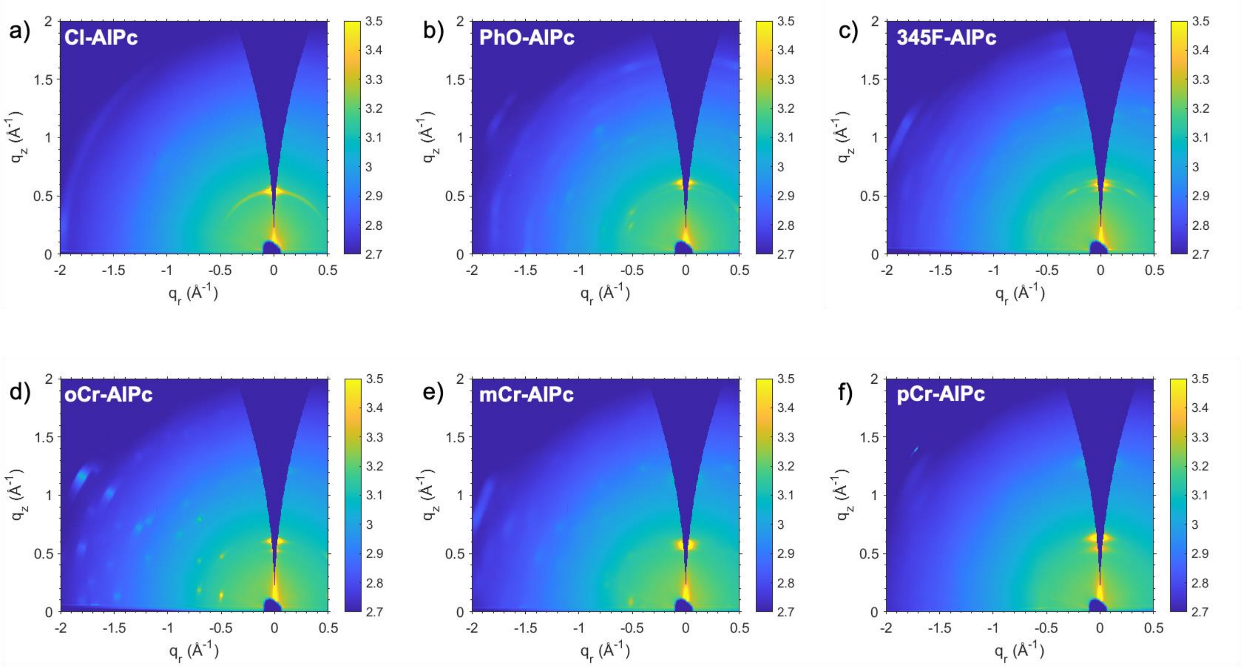

Figure S17. 2D GIWAXS spectra of (a) Cl-AlPc, (b) PhO-AlPc, (c) 345F-AlPc, (d) oCr-AlPc, (e) mCr-AlPc, and (f) pCr-AlPc films exposed to 300 ppm CBD vapor for 90 seconds.

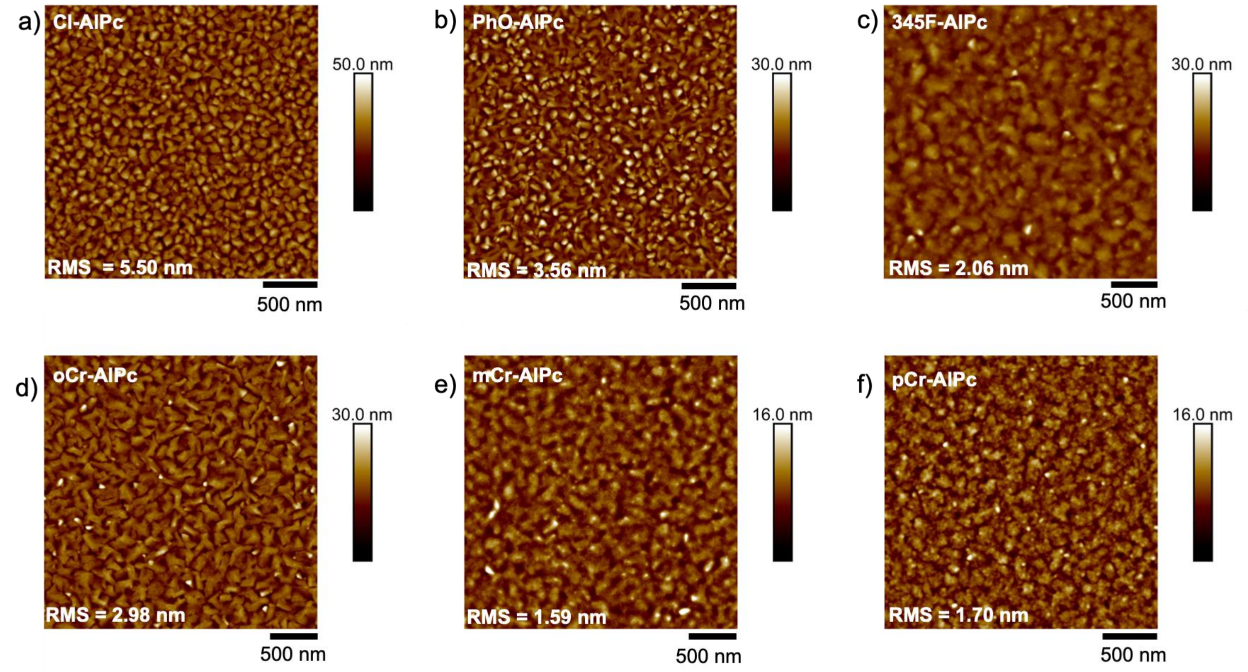

Figure S18. AFM images of (a) Cl-AlPc, (b) PhO-AlPc, (c) 345F_3-AlPc, (d) oCr-AlPc, (e) mCr-AlPc, and (f) pCr-AlPc, after exposure to 20 μM THC solution in hexanes. All images are 2.5 μm x 2.5 μm, with a scale bar of 500 nm.

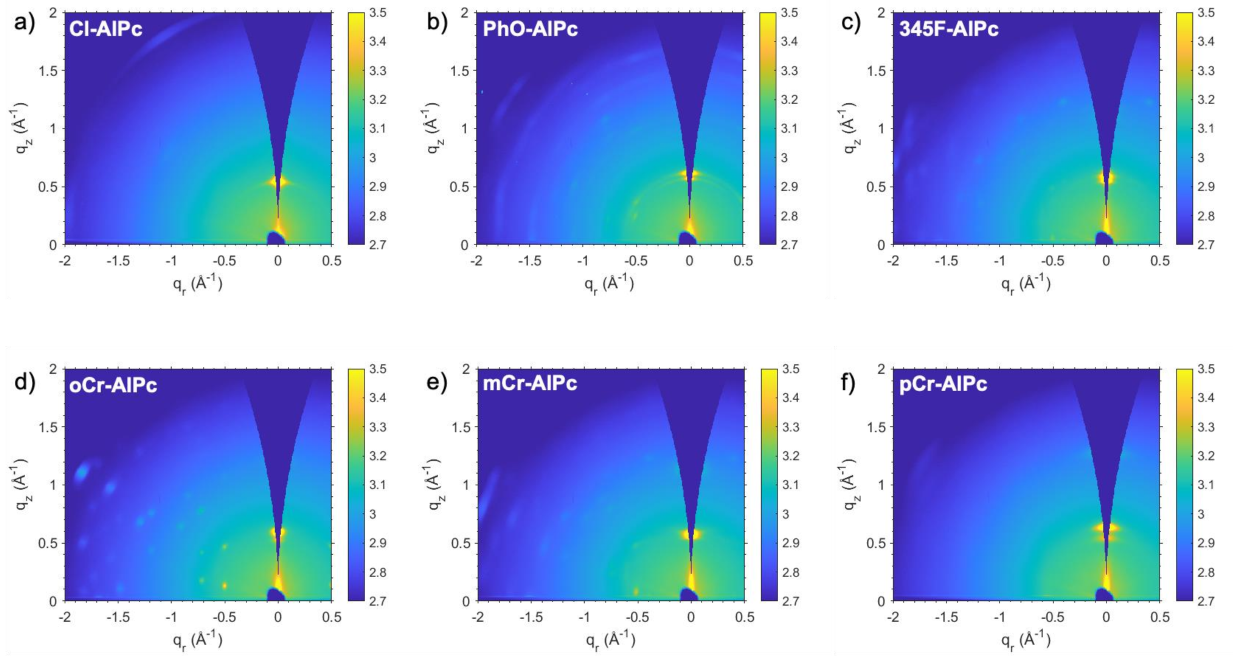

Figure S19. 2D GIWAXS spectra of (a) Cl-AlPc, (b) PhO-AlPc, (c) 345F-AlPc, (d) oCr-AlPc, (e) mCr-AlPc, and (f) pCr-AlPc films exposed to 20 μM THC solution in hexanes.

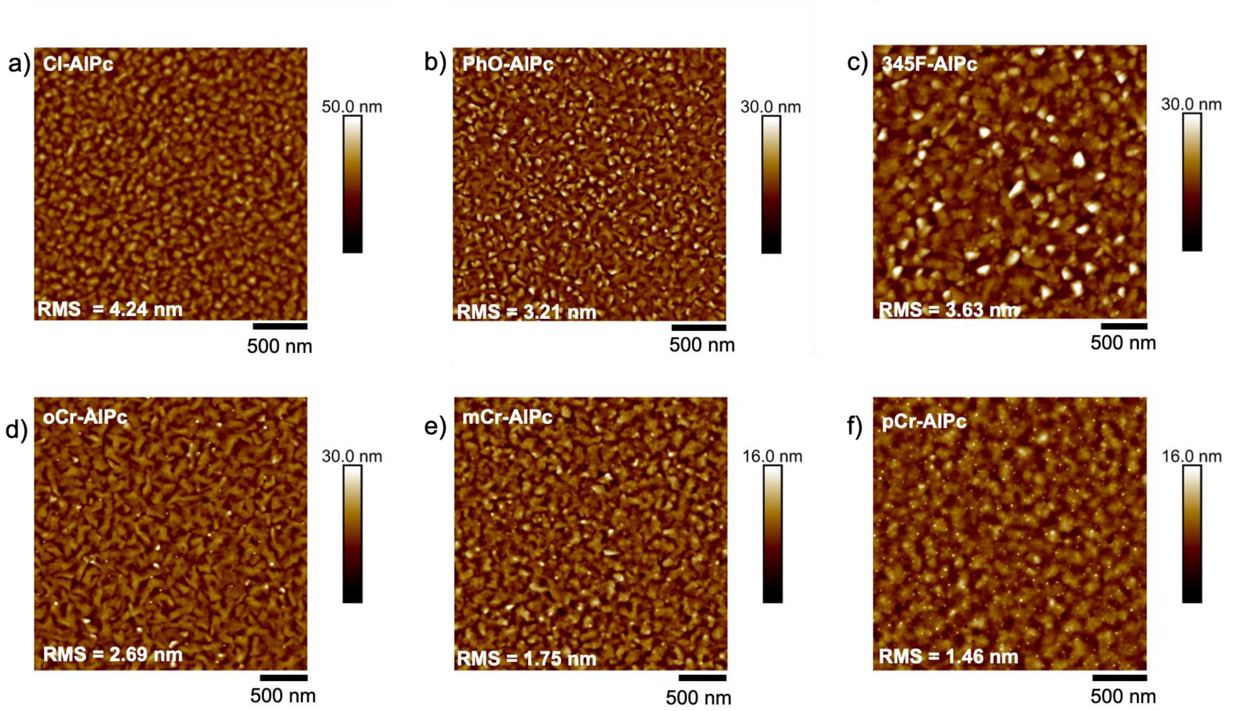

Figure S20. AFM images of (a) Cl-AlPc, (b) PhO-AlPc, (c) $345F_3$-AlPc, (d) oCr-AlPc, (e) mCr-AlPc, and (f) pCr-AlPc, after exposure to 20 μM CBD solution in hexanes. All images are 2.5 μm x 2.5 μm, with a scale bar of 500 nm.

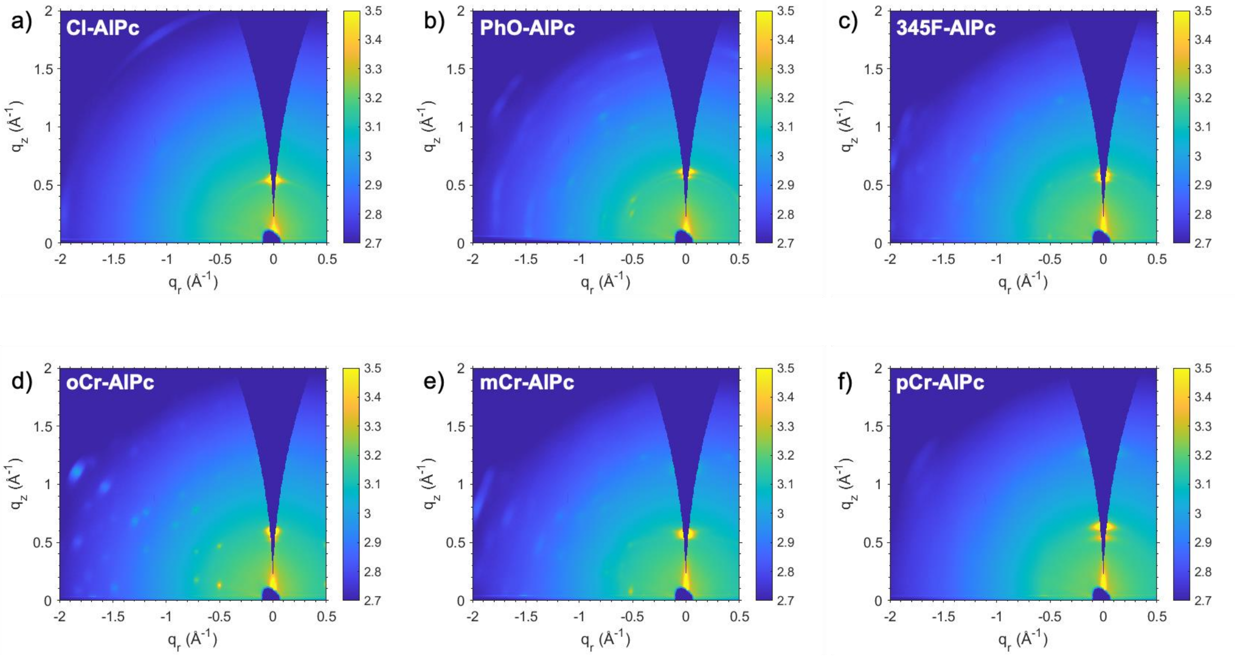

Figure S21. 2D GIWAXS spectra of (a) Cl-AlPc, (b) PhO-AlPc, (c) 345F-AlPc, (d) oCr-AlPc, (e) mCr-AlPc, and (f) pCr-AlPc films exposed to 20 μM CBD solution in hexanes.

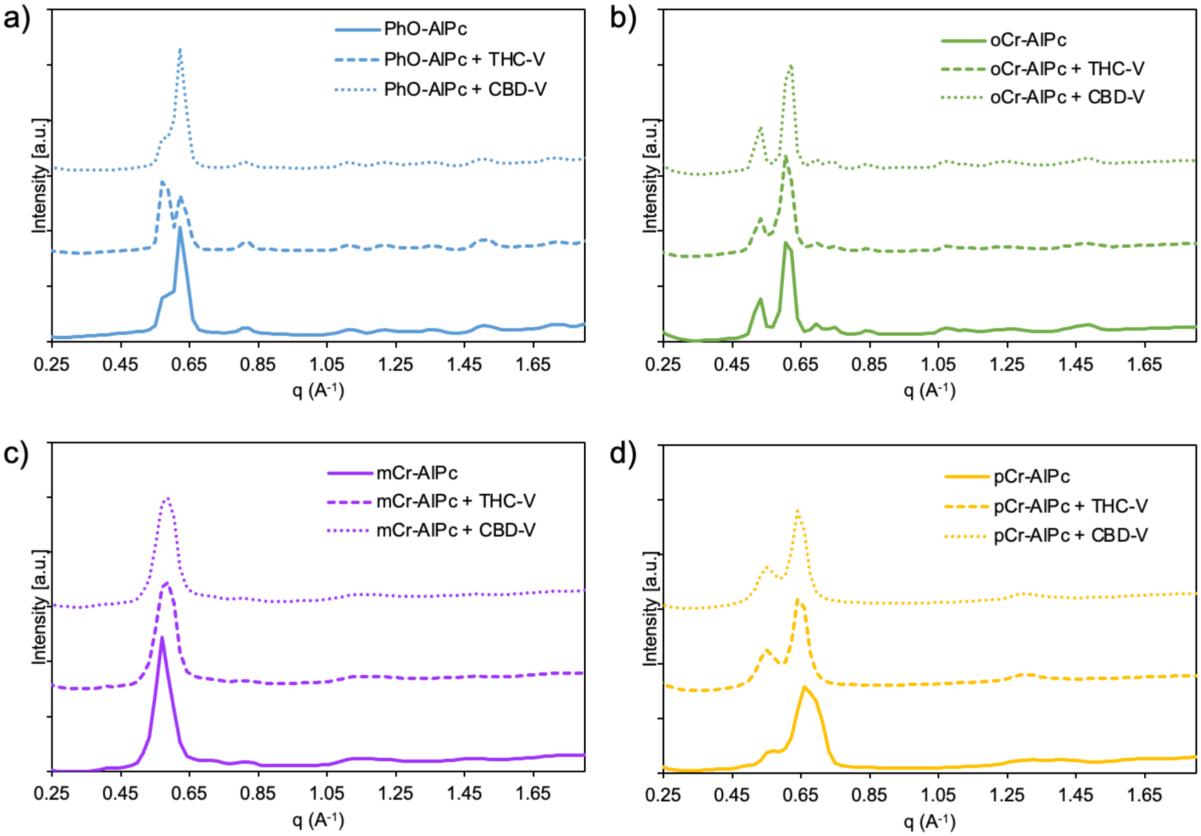

Figure S22. Diffraction pattern of (a) PhO-AlPc, (b) oCr-AlPc, (c) mCr-AlPc, and (d) pCr-AlPc, before and after exposure to 300 ppm THC vapor (THC-V) or 300 ppm CBD vapor (CBD-V), determined by GIWAXS.

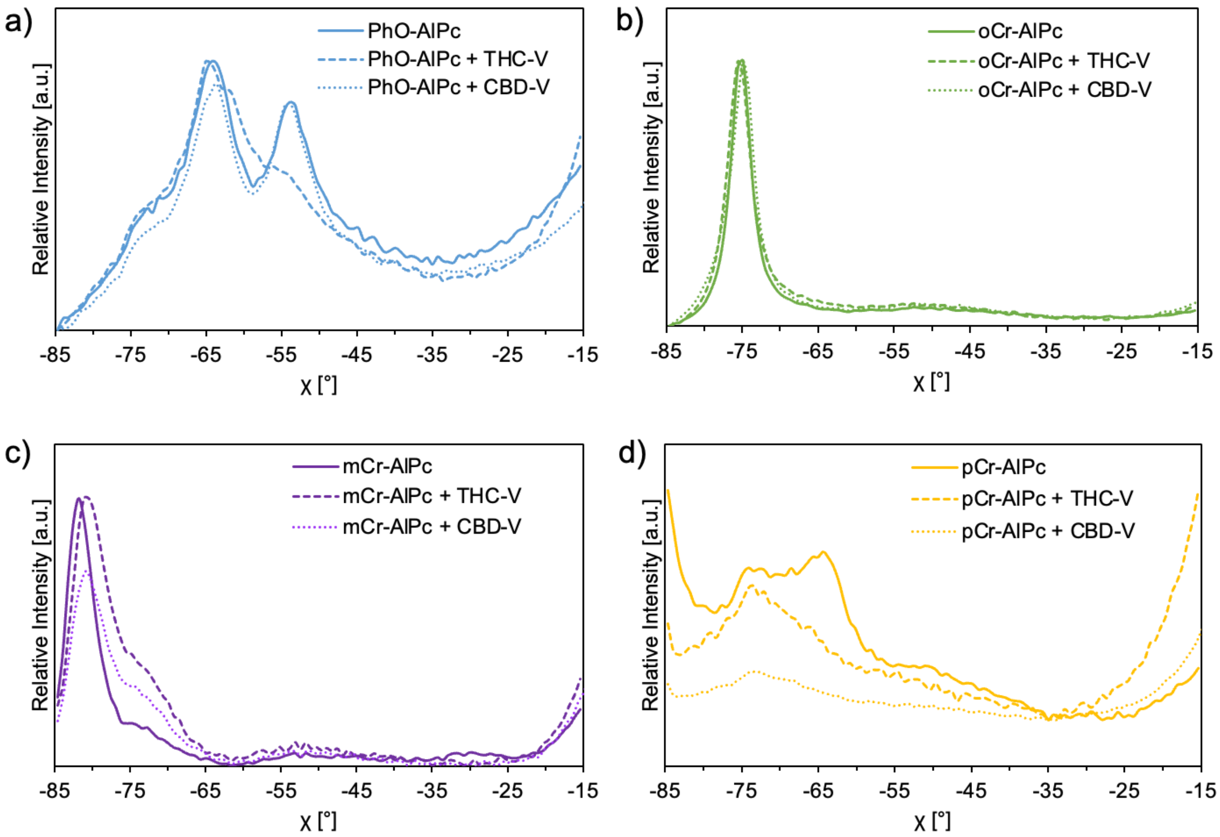

Figure S23. Linecut profiles with respect to χ using a *q* range of 0.55-0.65 Å^{-1} for (a) PhO-AlPc, (b) oCr-AlPc, (c) mCr-AlPc, and (d) pCr-AlPc before and after exposure to 300 ppm THC vapor (THC-V) or 300 ppm CBD vapor (CBD-V), determined by GIWAXS.

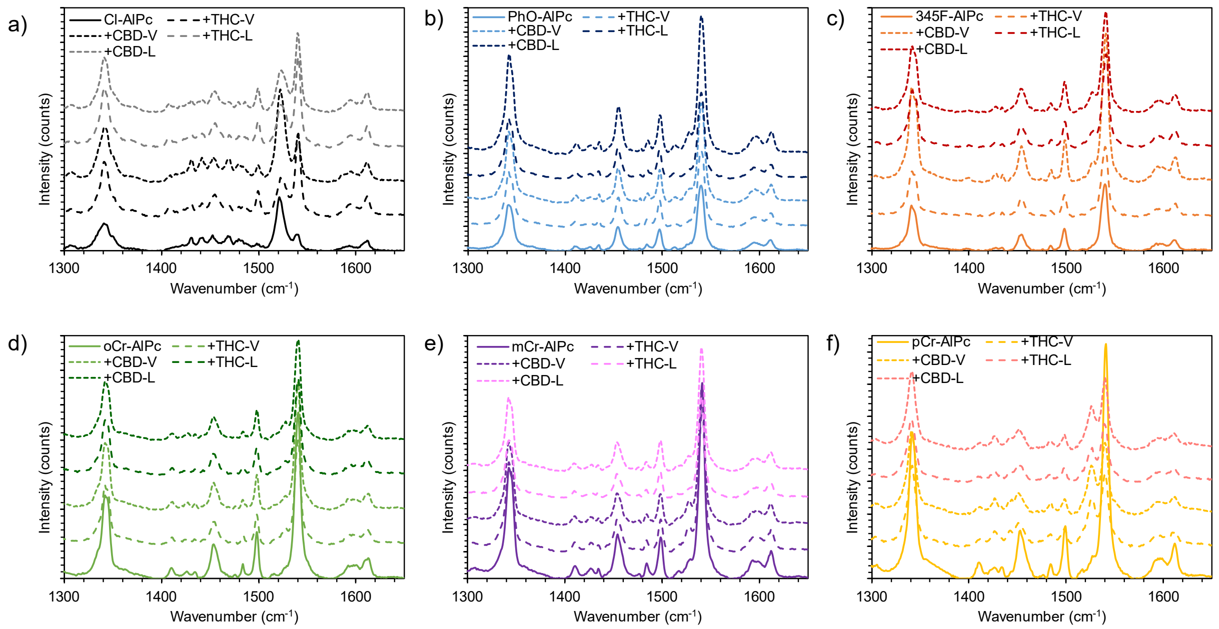

Figure S24. Raman spectra of (a) Cl-AlPc, (b) PhO-AlPc, (c) 345F-AlPc, (d) oCr-AlPc, (e) mCr-AlPc, and (f) pCr-AlPc before and after exposure to THC and CBD vapor and solution. The spectra were collected with a 532 nm laser at 10% power and 1-second exposure.

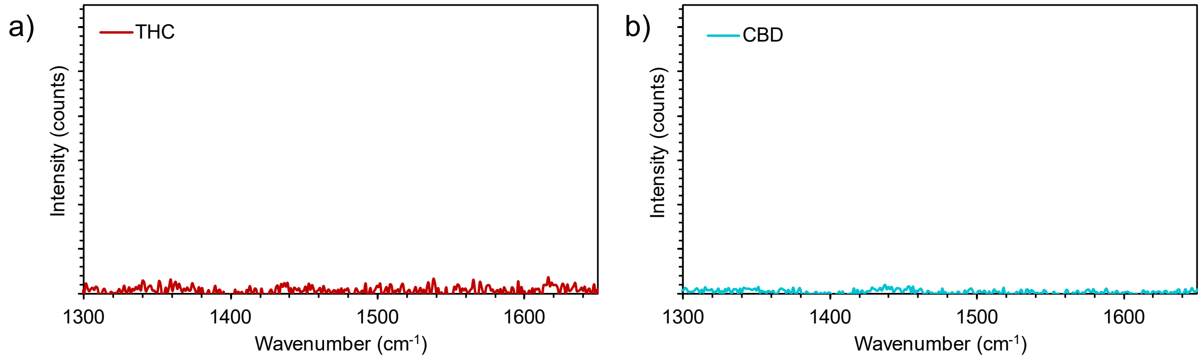

Figure S25. Raman spectra of pure (a) THC and (b) CBD oil collected by a 532 nm laser at 10% power and 5-second exposure.

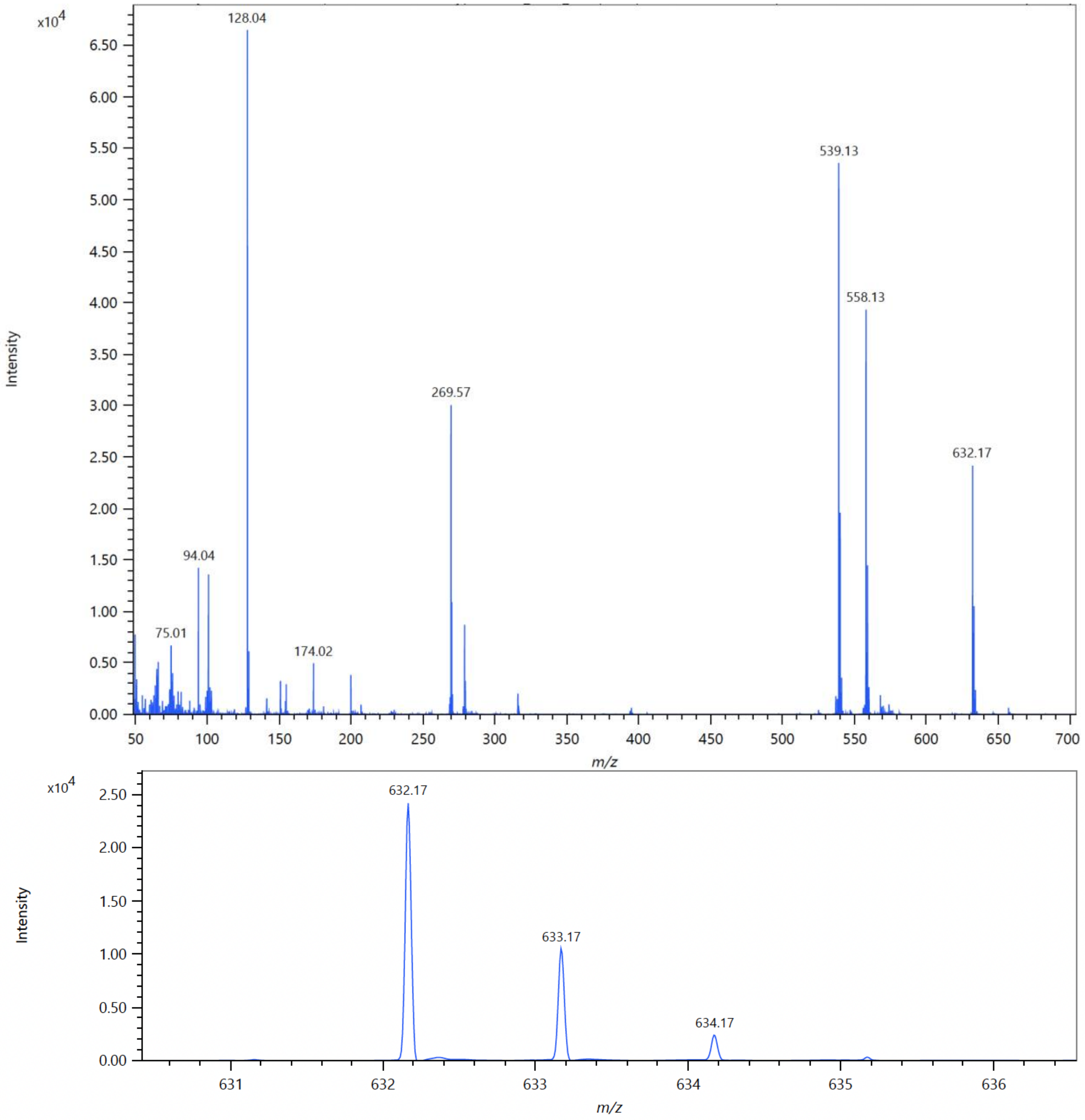

Elemental Composition

Parameters

		Elements Set 1:					
Tolerance:	±100.00 mDa	Symbol	C	H	N	O	Al
Electron:	Odd/Even	Min	0	0	0	0	1
Charge:	+1	Max	100	150	10	10	1
DBE:	-1.5 - 60.0						

Results

Mass	Intensity	Formula	Calculated Mass	Mass Difference [mDa]	Mass Difference [ppm]	DBE
632.16519	24169.05	C39 H27 N O6 Al	632.16483	0.36	0.58	27.5
		C38 H21 N8 O Al	632.16482	0.37	0.58	33.0
		C40 H23 N5 O2 Al	632.16616	-0.97	-1.54	32.5
		C26 H25 N10 O8 Al	632.16667	-1.48	-2.34	20.0
		C37 H25 N4 O5 Al	632.16348	1.71	2.70	28.0
		C42 H25 N2 O3 Al	632.16751	-2.32	-3.66	32.0

Figure S26. Low resolution (top) and high resolution mass spectrum (EI) of crude PhO-AlPc.

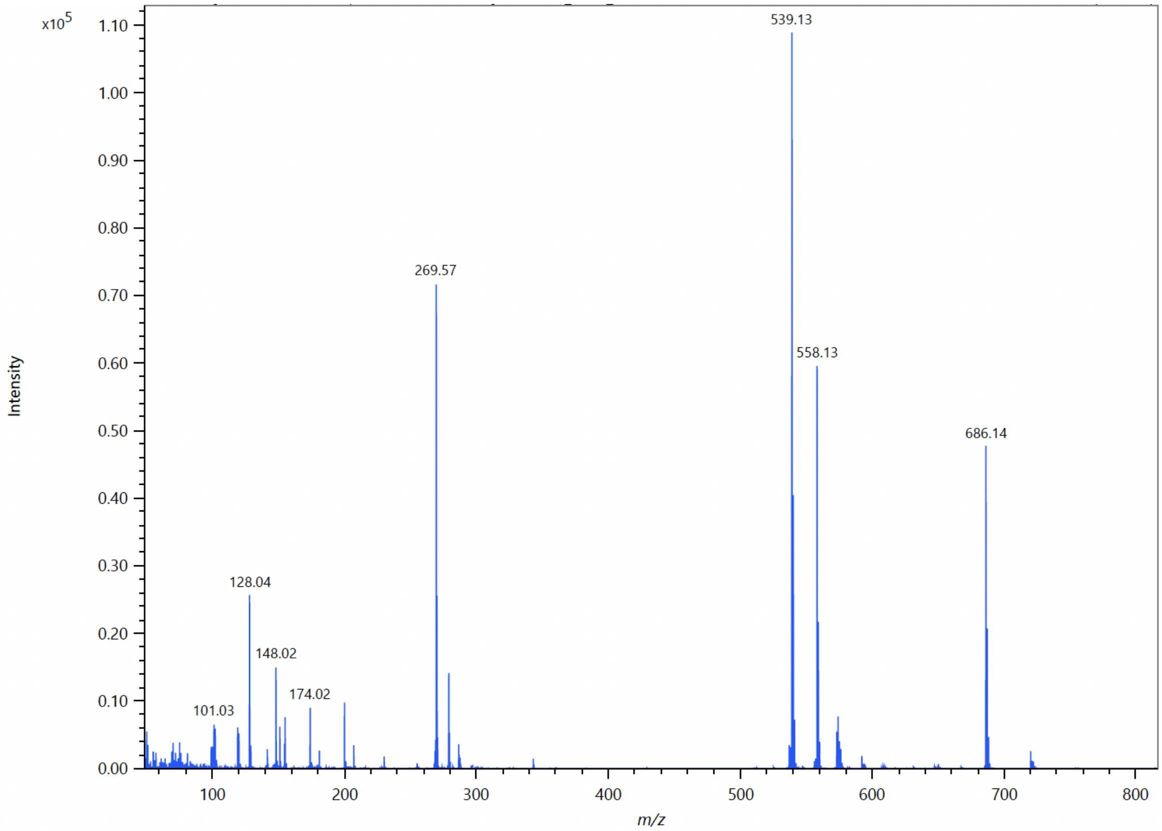

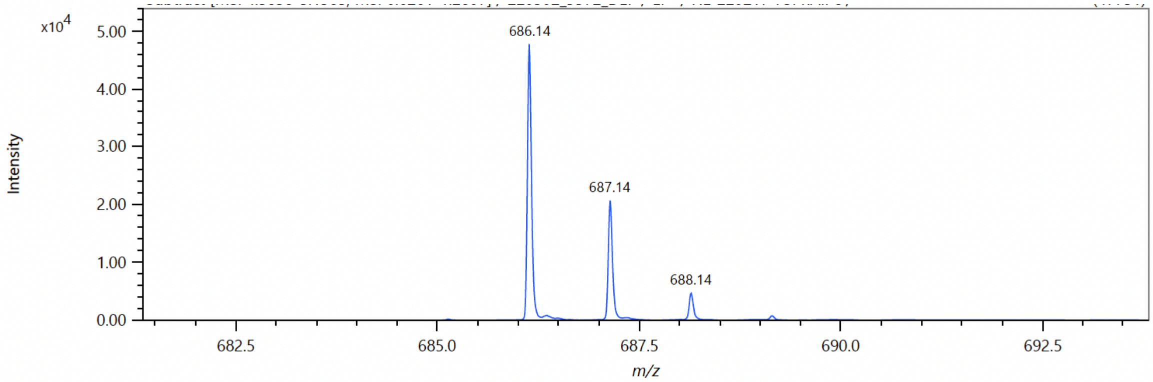

Elemental Composition

Parameters

Tolerance:	±40.00 mDa
Electron:	Odd/Even
Charge:	+1
DBE:	-1.5 - 60.0

Elements Set 1:

Symbol	C	H	N	O	Al	F
Min	0	0	0	0	1	3
Max	100	150	10	10	1	3

Results

Mass	Intensity	Formula	Calculated Mass	Mass Difference [mDa]	Mass Difference [ppm]	DBE
686.13704	47751.44	C39 H24 N O6 F3 Al	686.13656	0.48	0.70	27.5
		C38 H18 N8 O F3 Al	686.13656	0.48	0.71	33.0
		C40 H20 N5 O2 F3 Al	686.13790	-0.86	-1.25	32.5
		C26 H22 N10 O8 F3 Al	686.13841	-1.37	-1.99	20.0
		C37 H22 N4 O5 F3 Al	686.13522	1.82	2.66	28.0
		C42 H22 N2 O3 F3 Al	686.13924	-2.20	-3.21	32.0

Figure S27. Low resolution (top) and high resolution (bottom) mass spectrum (EI) of crude 345F-AlPc.

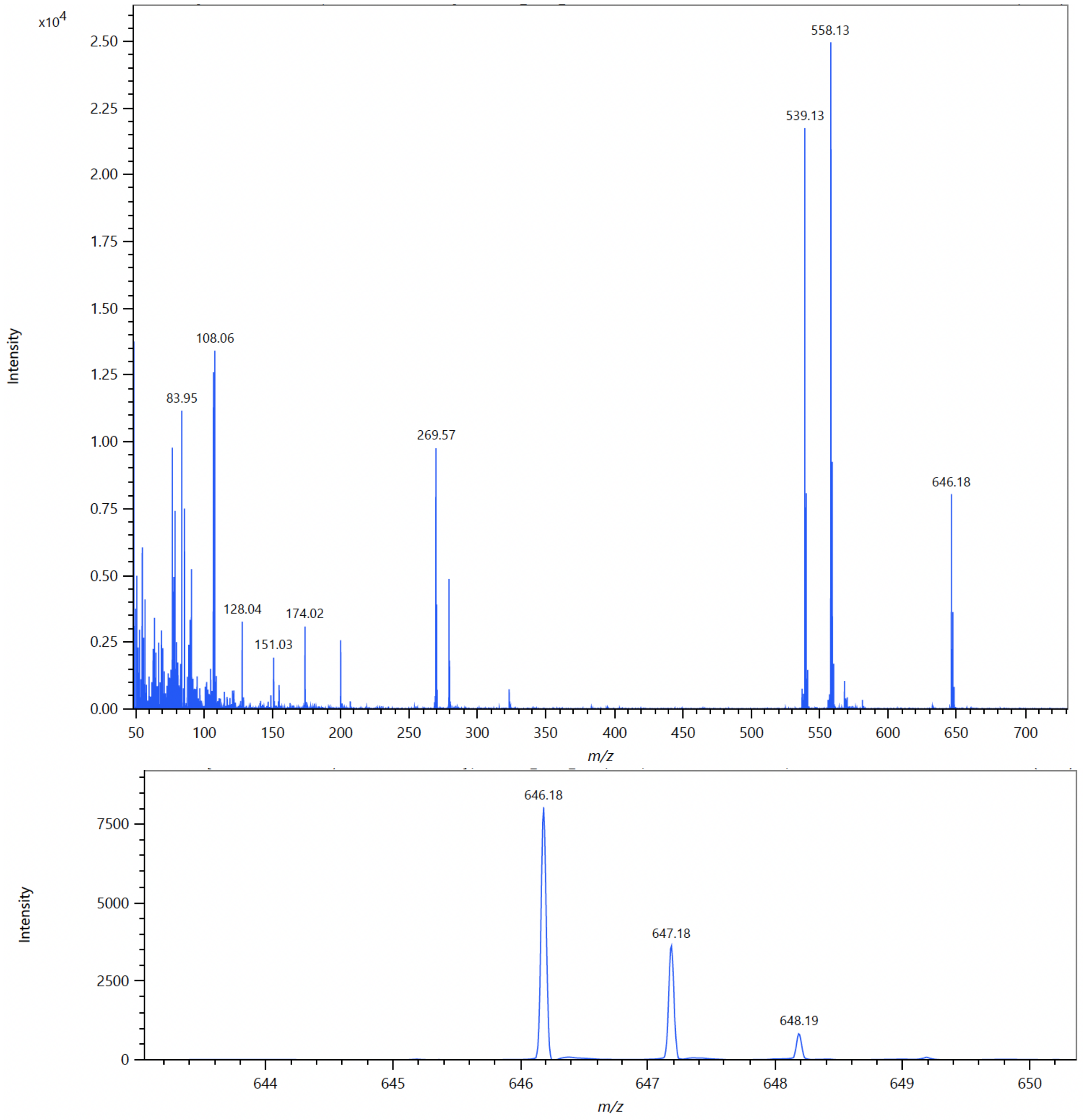

Elemental Composition

Parameters

Tolerance:	±100.00 mDa
Electron:	Odd/Even
Charge:	+1
DBE:	-1.5 - 60.0

Elements Set 1:

Symbol	C	H	N	O	Al
Min	0	0	0	0	1
Max	100	150	10	10	1

Results

Mass	Intensity	Formula	Calculated Mass	Mass Difference [mDa]	Mass Difference [ppm]	DBE
646.18154	8042.70	C41 H25 N5 O2 Al	646.18181	-0.28	-0.43	32.5
		C27 H27 N10 O8 Al	646.18232	-0.79	-1.22	20.0
		C40 H29 N O6 Al	646.18048	1.06	1.64	27.5
		C39 H23 N8 O Al	646.18047	1.07	1.65	33.0
		C43 H27 N2 O3 Al	646.18316	-1.62	-2.51	32.0
		C29 H29 N7 O9 Al	646.18367	-2.13	-3.29	19.5

Figure S28. Low resolution (top) and high resolution (bottom) mass spectrum (EI) of crude oCr-AlPc

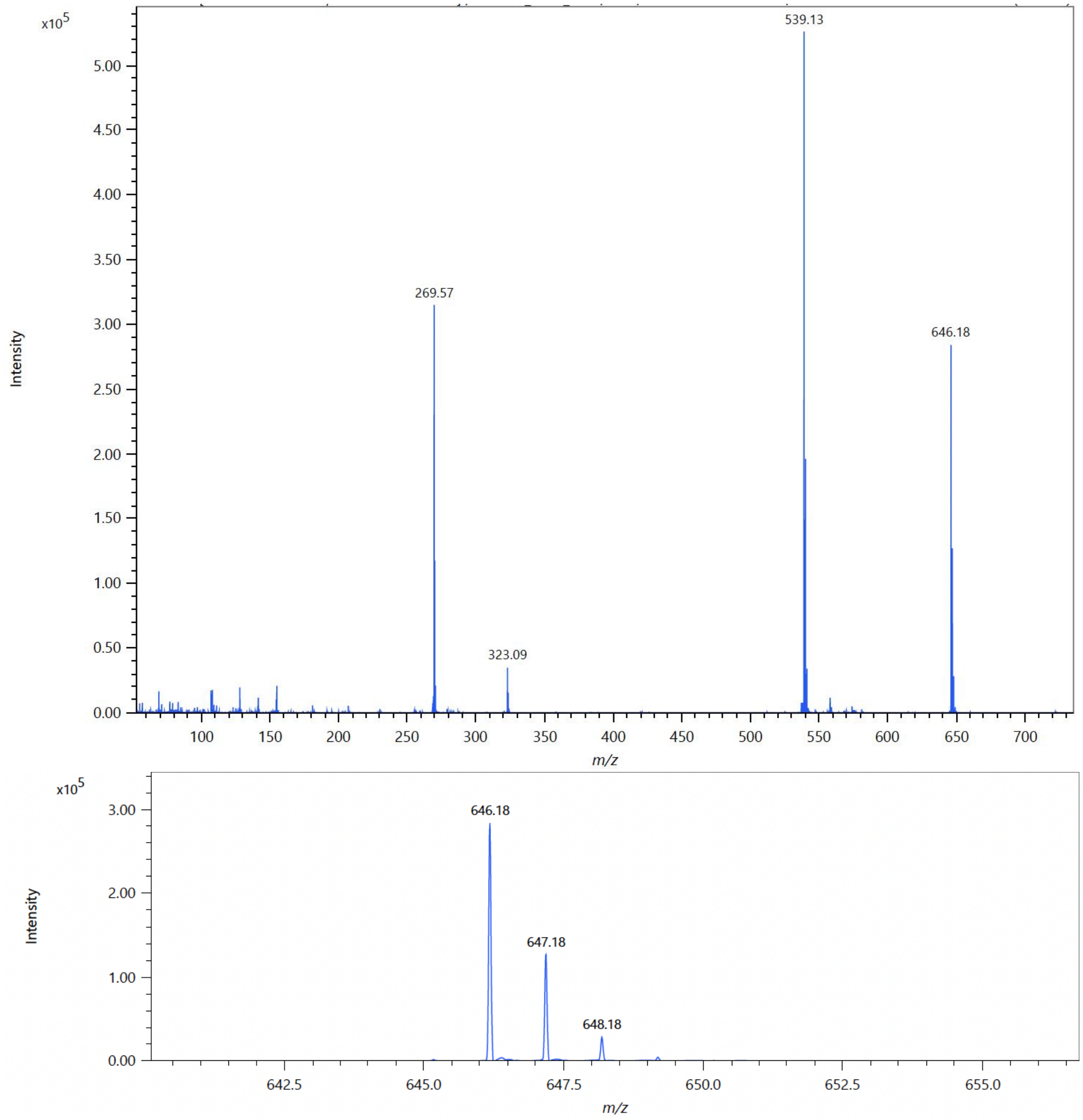

Elemental Composition

Parameters		Elements Set 1:					
		Symbol	C	H	N	O	Al
Tolerance:	±100.00 mDa						
Electron:	Odd/Even	Min	0	0	0	0	1
Charge:	+1	Max	100	150	10	10	1
DBE:	-1.5 - 60.0						

Results

Mass	Intensity	Formula	Calculated Mass	Mass Difference [mDa]	Mass Difference [ppm]	DBE
646.17852	283989.92	C38 H27 N4 O5 Al	646.17913	-0.62	-0.96	28.0
		C37 H31 O9 Al	646.17780	0.72	1.11	23.0
		C36 H25 N7 O4 Al	646.17779	0.73	1.12	28.5
		C39 H23 N8 O Al	646.18047	-1.95	-3.03	33.0
		C40 H29 N O6 Al	646.18048	-1.96	-3.03	27.5
		C35 H29 N3 O8 Al	646.17645	2.06	3.19	23.5

Figure S29. Low resolution (top) and high resolution (bottom) mass spectrum (EI) of crude mCr-AlPc.

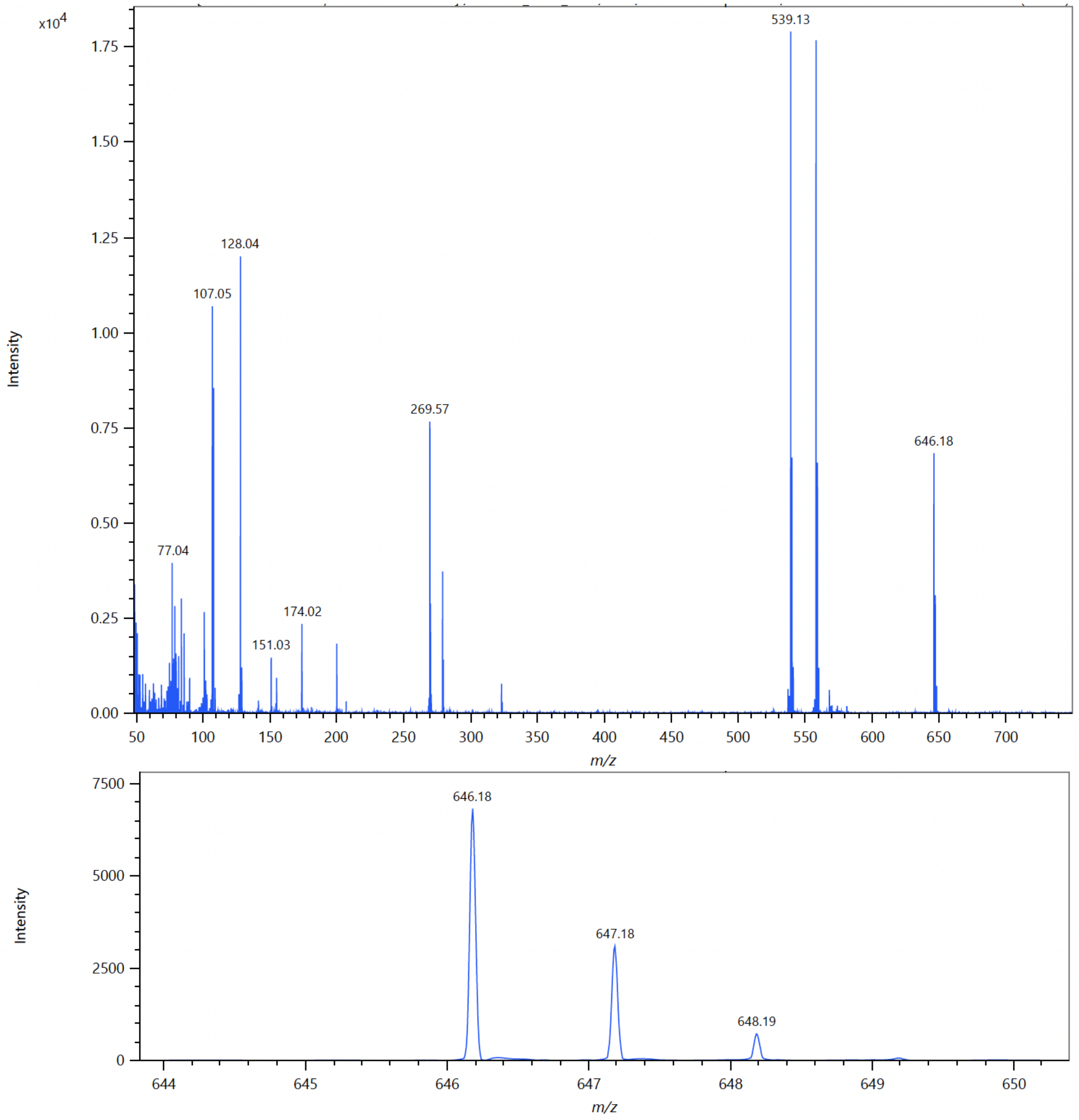

Elemental Composition

Parameters		Elements Set 1:					
Tolerance:	±100.00 mDa	Symbol	C	H	N	O	Al
Electron:	Odd/Even	Min	0	0	0	0	1
Charge:	+1	Max	100	150	10	10	1
DBE:	-1.5 - 60.0						

Results

Mass	Intensity	Formula	Calculated Mass	Mass Difference [mDa]	Mass Difference [ppm]	DBE
646.18114	6827.35	C40 H29 N O6 Al	646.18048	0.66	1.02	27.5
		C39 H23 N8 O Al	646.18047	0.67	1.03	33.0
		C41 H25 N5 O2 Al	646.18181	-0.68	-1.05	32.5
		C27 H27 N10 O8 Al	646.18232	-1.18	-1.83	20.0
		C38 H27 N4 O5 Al	646.17913	2.00	3.10	28.0
		C43 H27 N2 O3 Al	646.18316	-2.02	-3.12	32.0

Figure S30. Low resolution (top) and high resolution (bottom) mass spectrum (EI) of crude pCr-AlPc
